# Single Molecule Magnets of Co_2_ and Co_2_La MOFs Synthesized by New Schiff Base Ligand N,N′-bis(*o*-Vanillinidene) Ethylenediamine (*o*-VEDH_2_)

**DOI:** 10.3389/fchem.2020.571223

**Published:** 2020-11-12

**Authors:** Mithun Kumar Ghosh, Barun Jana, Tanmay Kumar Ghorai

**Affiliations:** ^1^Nanomaterials and Crystal Designing Laboratory, Department of Chemistry Indira Gandhi National Tribal University, Amarkantak, India; ^2^Department of Chemistry, Malaviya National Institute of Technology, Jaipur, India; ^3^Nanomaterials and Crystal Designing Laboratory, Department of Chemistry, Guru Ghasidas Vishwavidyalaya, Bilaspur, India

**Keywords:** synthesis, schiff base, MOFs, SMMs, sensing and CT-DNA 3

## Abstract

Schiff base ligand N,N′-bis(*o*-vanillinidene) ethylenediamine (*o*-VEDH_2_) has been employed to synthesize new [Co^II^Co^IV^(*o*-VED)(OAc)_2_(μ_2_-OAc)(OMe)]•MeOH (**1**) and [${\text{Co}}_{2}^{\text{IV}}$(*o*-VED)_2_(en)_2_(NCCH_3_)(OCH_3_)][La(NO_3_)_6_](${\text{NO}}_{3}-$)•2MeOH•MeCN•H_2_O (**2**) metal–organic frameworks (MOFs) that have interesting single molecule magnets (SMMs) property. The synthesized complexes are characterized by single crystal X-ray diffraction, fourier transform infrared spectroscopy (FTIR), UV-visible spectroscopy, and squid magnetic measurement. Single crystal X-ray data show that both complexes crystallize in the monoclinic crystal system with *P*2_1_/*c*(14) and *P*2_1_/*n*(14) space groups and generate unique MOF-like structures. Overall, both the metal centers of **1** form octahedral geometry with a butterfly core structure. Variable temperature (*T*) and field (*H*) solid-state direct-current (dc) and alternative current (ac) magnetic susceptibility measurements were performed on both the complexes over 1.8 to 300 K, which exhibited a ground state spins (S) of 4 and 5 of complexes **1** and **2**, respectively. The AC out-phase and in-phase properties of complexes show SMMs. Other properties such as optical, sensing, and DNA-binding interactions were also investigated by the complexes. Complexes **1** and **2** have energy band gaps of 3.7 and 3.03 eV indicating semiconductor properties. Simultaneously, complex **1** was found to sense H_2_O_2_ with a rate constant (*k*) = 1.59 × 10^−4^ s^−1^, whereas complex **2** was found to bind with calf-thymus–DNA by intercalation mode with binding constant (*K*_*b*_) of 1.22 × 10^5^ M^−1^.

## Introduction

In the last few years, multifunctional homo/pseudoheterometallic metal–organic frameworks (MOFs) showing distinct physical properties have been reported not only for the basic intensive research but also for their potential applications in new technologies (Yang et al., 2015; Zhang et al., 2016; Clough et al., 2017; Dong et al., 2017; Long, 2019; Qi et al., 2019). In material science, multifunctional metal complexes having magnetomolecular properties are of interest because they found potential applications in the field of sensors and biology (Chelebaeva et al., 2009; Montgomery et al., 2009; Yuan et al., 2018). In recent literatures, different groups have reported the syntheses of different bimetallic complexes with bifunctional magnetomaterial properties (Freitas et al., 2010; Kumar et al., 2019; Wu et al., 2019). Here, the magnetic properties of single molecules are of interest, and significant amount of results is available (Sessoli et al., 1993; Gatteschi and Sessoli, 2003; Tasiopoulos et al., 2004; Bogani and Wernsdorfer, 2010; Van Slageren, 2011; Vincent et al., 2012; Wu et al., 2019). The chemistry of single molecule magnets (SMMs) was started through the discovery of Mn_12_Ac in 1993. It was found that large ground state spin (S) and negative zero field splitting (D) are the two essential factors that determine SMMs properties (Sessoli et al., 1993; Gatteschi and Sessoli, 2003; Tasiopoulos et al., 2004; Bogani and Wernsdorfer, 2010; Van Slageren, 2011; Vincent et al., 2012; Wu et al., 2019). In this regard, syntheses of lanthanide-based SMMs are quite of interest because of their various potential applications in high-density storage, quantum computers, and spintronics devices (Sessoli et al., 1993; Ishikawa et al., 2003; King et al., 2005; Tang and Zhang, 2015; Ding et al., 2016). Along with this, the mixed cobalt and lanthanide complexes with Schiff based ligands are also of significant interest because of their SMMs properties (Gupta et al., 2016; Modak et al., 2016). At the same time, metal complexes of Schiff base ligands are also of huge importance in the field of medicinal and pharmaceutical chemistry due to their antibacterial (Woodruff et al., 2013), antifungal (Lv et al., 2006), and antitumor activities (Alghool et al., 2013) and DNA-binding (Yang et al., 2000) properties. Effective anticancer drugs generally have an active site that mainly binds to the cancer cell's DNA and reduces cancer cell activity, which lead to death of the cells (Wang et al., 2006, 2011; Pathak et al., 2018). Medicines that are used to prevent cancer from interacting with DNA through electrostatic, intercalative, or groove binding depend on binding methods of anticancer drugs (Zuber et al., 1998; Li et al., 2010; Pages et al., 2015; Zeng et al., 2017). In the past few years, researchers have reported cobalt and lanthanum complexes of Schiff base ligands and investigated their calf-thymus (CT)–DNA binding (Yang et al., 2006; Santini et al., 2013; Wang et al., 2014).

In the biological field, it is well-known that reactive oxygen species (ROS) and reactive nitrogen species play a significant role in the development of cancer cells in the human body. ROS such as superoxide anion (${\text{O}}_{2}^{•-}$) and hydroxyl radical (•OH) are generally generated during the decomposition of hydrogen peroxide, which is the main reason for cancer cell generation (Tredwin et al., 2006; Shahabadi et al., 2010; Zhao et al., 2012). The development of practical and accurate H_2_O_2_ detection techniques is therefore necessary to ensure improved healthcare. Last few years, scientists have developed lanthanide/transition metal–based MOFs and investigated their different sensing activity (Droge, 2002; Gao et al., 2016; Koo et al., 2016; Zeng et al., 2016). Herein, we report the H_2_O_2_-sensing activity of Co-MOFs.

Our research group focused on the synthesis of magnetomolecular materials that have multifunctional applications. Recently, we have reported magnetomolecular Cu_3_ 1D polymer and Mn_12_ benzoate cluster that have multifunctional applications such as antibacterial, anticancer, antioxidant, cytotoxicity, and magnetic (Mandal et al., 2017; Pathak et al., 2019). From the literature examples, it is clear that multifunctional homometallic Co/Co or pseudoheterometallic Co/La clusters might show properties relevant to sensing, biological, and magnetic properties. Herein, we report two new homometallic and pseudoheterometallic Co and Co-La–based metal clusters along with their SMMs, optical, sensing, and DNA-binding interaction properties.

## Results and Discussions

For the synthesis of pseudoheterometallic Co/La metal cluster, we have chosen the simple N,N′-bis(*o*-vanillinidene) ethylenediamine (*o*-VEDH_2_) as our ligand system. Here, the presence of two different bonding atoms (O, N) may preferentially choose one above the other between Co and La, and that may result in the formation of our desired product. Accordingly, we have reacted to the readily synthesized (*o*-VEDH_2_) with a mixture of La(NO_3_)_3_·6H_2_O and Co(NO_3_)_2_·6H_2_O in methanol in 2:1:1 ratio. Subsequent work and crystallization resulted in the formation of black crystals. Elemental analysis and infrared spectroscopy indicated the formation of the pseudoheterometallic cluster (**2**). Determination of molecular structure via single crystal X-ray diffraction further confirmed the formation of a pseudoheterometallic Co/La metal cluster. For comparison purposes, we have also decided to synthesize a homometallic Co/Co molecular cluster via a 1:2 reaction of (*o*-VEDH_2_) and Co(OAc)_2_ in methanol. In this case, subsequent workup also led to the isolation of black crystals. Initial characterization utilizing elemental analysis and infrared spectroscopic data indicated the formation of a homometallic bis-Co cluster (**1**). The molecular geometry of cluster **1** was established via a single crystal X-ray diffraction study.

### Structural Description

**Figure 1:**
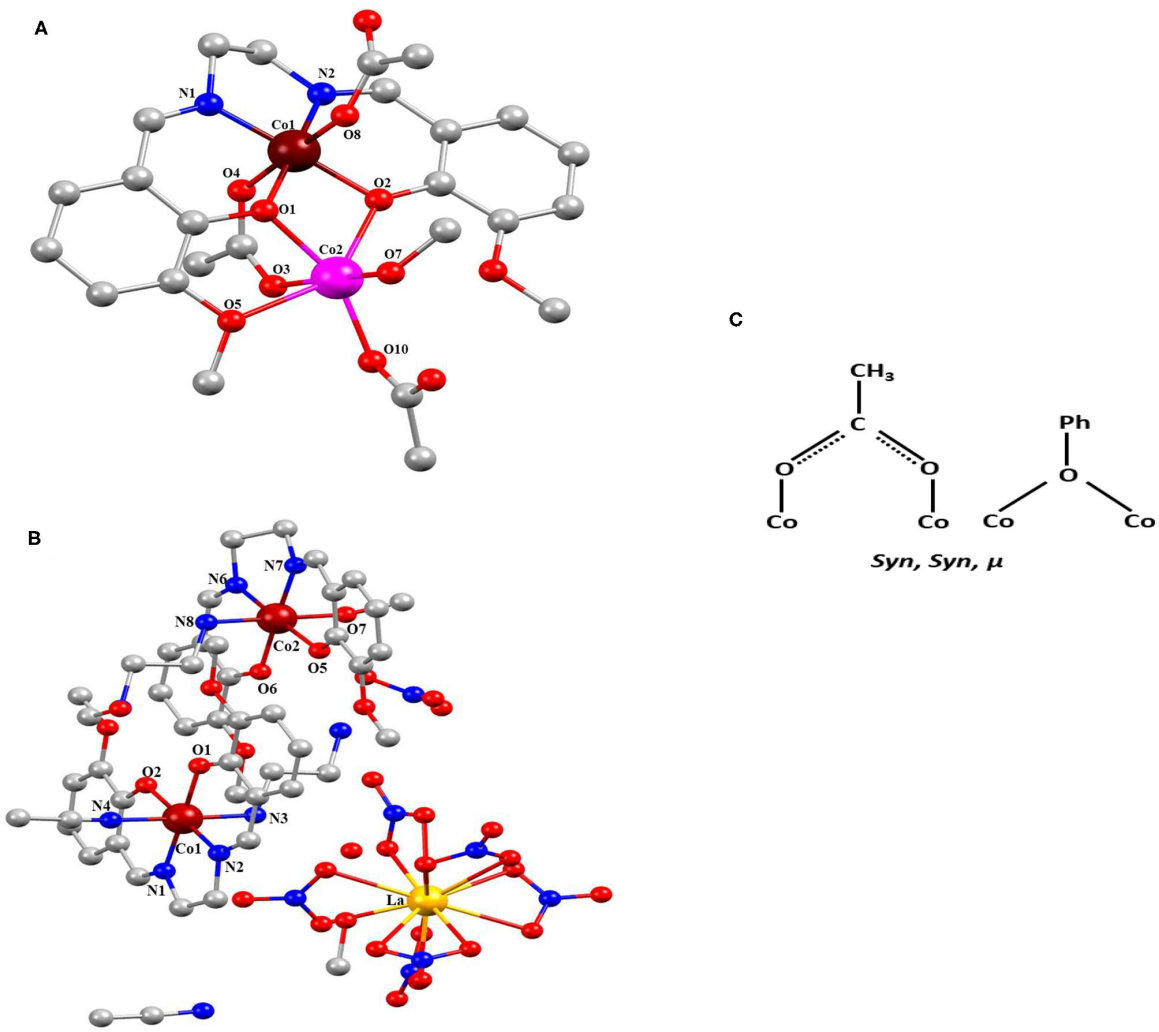
**(A)** Complex 1 and **(B)** complex 2 [pink, Co(II); blackish brown, Co(IV); yellow, La; red, O; blue, N; gray, C and H removed for clear structure]. **(C)** Coordination modes of complexes 1 and 2.

Complexes **1** and **2** are crystalized in the monoclinic system with *P*2_1_/*c* and *P*2_1_/*n*. Figure 1 shows molecular structures of **1** and **2**. Crystal data and selected bond distances and angles are summarized in Tables 1, 2, respectively. In complex 1, two cobalt atoms are residing in two different electronic environments and surrounded by one *o*-VED ligand moiety, coordinated through both the oxygen and nitrogen atom, and OAc^−^ anions either through μ_2_ or μ_3_ coordination mode. Both *o*-VED ligand nitrogen (N1 and N2), coordinated with the Co1 atom and *o*-VED's two hydroxo-oxygen (O1 and O2) bridged with both the Co1 and Co2. Further, one of the acetate anions (O3 and O4) bridges both the Co atoms in a μ_2_ coordination mode. The octahedral coordination geometry of the two Co atoms has been achieved by the addition of terminally connected acetate groups via O8 and O10, respectively. Overall, both the metal centers form octahedral geometry with a butterfly core structure. The distance between Co1 and Co2 is 3.043(19) Å, which is relatively long for any covalent interaction among them. Interestingly, the average bond distance from Co1 to its two coordinating nitrogen atoms is 1.866 Å, whereas the average bond distance to its four coordinating oxygen atoms is 1.902 Å. Relatively short bond distances between Co-O and Co-N indicate that the Co atom may be present in +4 oxidation state. On the other hand, the average bond distance from Co2 atom to its six coordination oxygen atoms is 2.079Å. Relatively higher Co-O bond lengths indicate that the Co2 present in a lower oxidation state as compared to Co1. Literature references indicate that in this case, Co2 may present in +2 oxidation state. Interestingly, complex **2** is a double salt of Co_2_ and La fragments. In complex **2**, both the Co atoms are coordinated to two separate *o*-VED moieties through their oxygen atoms (O_1_ and O_2_ to Co_1_; O_5_ and O_6_ to Co_2_) and nitrogen atoms (N1 and N2 to Co1; N6 and N7 to Co2). In addition to this, both the central cobalt atoms are terminally coordinated to two different ethylene diamine groups through one of the nitrogen of –NH_2_ groups (N3 to Co1 and N8 to Co2). The octahedral coordination sphere of Co1 is completed via coordination of an additional acetonitrile group (N4), whereas the octahedral coordination of Co2 gets fulfilled via coordination of an –OMe group (O7). The anionic part is the nitrate coordinated lanthanum complex where six nitrate ions are coordinated to a single La atom. The nearest distances between Co1…Co2, Co1…La1, and Co2…La1 were 7.215 (11), 7.335 (10), and 9.497 (11) Å, respectively. The plausible coordination modes of complexes **1** and **2** are shown in Figure 1C.

**Table 1:** Phase data of complexes 1 and 2.

**Parameter**	**Complex 1**	**Complex 2**
Formula	C_26_ H_34_ Co_2_ N_2_ O_12_	C_47_H_58_Co_2_LaN_17_O_33_
Formula weight (g mol^−1^)	684.41 g/mol	1,645.87 g/mol
Temperature (K)	101 K	100 K
Wavelength	0.71073 Å	0.71073 Å
Crystal system	Monoclinic	Monoclinic
Space group	*P*2_1_/*c* (14)	*P*2_1_/*n* (14)
Unit cell dimensions *a, b, c* (Å)	*a* = 8.9157 (14) Å, *b* = 13.359 (2) Å, *c* = 24.066 (4) Å	*a* = 12.8836 (11) Å, *b* = 19.6476 (17) Å, *c* = 25.664 (2) Å
α, β, γ (°)	α = 90, β = β = 90.602(6)°, γ = 90	α = 90, β = 94.574(3)°, γ = 90
*V* (Å^3^)	2,866.22 (80) Å^3^	6,475.69 (90) Å^3^
*Z*	4	4
Density (g/cm^3^)	1.58595 g/cm^3^	1.68808 g/cm^3^
Radiation type	Mo Kα	Mo Kα
μ (mm^−1^)	1.192	1.260
Absorption correction	Multiscan absorption correction	Multiscan absorption correction
*T*min, *T*max	0.867,0.898	0.970, 0.975
*R*int	0.0835	0.1448
Theta (max) Ǎ^−3^)	71.360	27.115
Refinement *R*[*F*^2^> 2σ(*F*^2^)], *wR*(*F*^2^), *S*	0.1239 (4,271), 0.3538 (4,562), 1.260	0.0565 (9,755), 0.1762 (14,277), 0.888
No. of reflections	4,562	14,277
No. of parameters	388	912
Data completeness	0.902	0.997

*R1 = Σ(||Fo| – |Fc||) / Σ|Fo|*.

*wR2 = [Σ[w(Fo^2^ – Fc^2^)^2^] / Σw[(Fo^2^)^2^]]^1/2^*.

*S = [Σ[w(Fo^2^ – Fc^2^)^2^] / (n-p)]^1/2^*.

w = 1/[Σ^2^(Fo^2^) + (m * p)^2^ + n * p], p = [max(Fo^2^,0)+ 2 * Fc^2^]/3, m and n are constants.

**Table 2:** Selected bond distances (Å) and bond angles (°) for complexes 1 and 2.

**Bond length**					
**Complex 1**		**Complex 2**			
**Bond type**	**Bond length (Å)**	**Bond type**	**Bond length (Å)**	**Bond type**	**Bond length (Å)**
Co1-N2	1.856 (8)	La1-O24	2.585 (4)	Co1-N1	1.878 (5)
Co1-N1	1.876 (8)	La1-O14	2.604 (4)	Co1-N2	1.889 (4)
Co1-O8	1.892 (7)	La1-O29	2.613 (4)	Co1-O2	1.901 (4)
Co1-O2	1.895 (7)	La1-O27	2.625 (4)	Co1-O1	1.916 (4)
Co1-O1	1.902 (7)	La1-O23	2.651 (4)	Co1-N3	1.941(4)
Co1-O4	1.920 (7)	La1-O26	2.651 (4)	Co1-N4	1.944 (5)
Co2-O10	2.004 (8)	La1-O21	2.652 (4)	Co2-N7	1.876 (4)
Co2-O7	2.065 (8)	La1-O18	2.654 (4)	Co2-N6	1.884 (4)
Co2-O5	2.14 (8)	La1-O20	2.659 (4)	Co2-O6	1.911 (4)
Co2-O3	2.062 (8)	La1-O30	2.662 (4)	Co2-O5	1.914 (4)
Co2-O1	2.069 (7)	La1-O17	2.663 (4)	Co2-N8	1.950 (4)
Co2-O2	2.137 (7)	La1-O15	2.749 (4)	Co2-O7	1.973 (4)
**Bond angle**					
**Bond type**	**Angle (°)**	**Bond type**	**Angle (°)**	**Bond type**	**Angle (°)**
N2-Co1-N1	86.0 (4)	N1-Co1-N2	84.7 (2)	O24-La1-O14	175.53 (14)
N2-Co1-O8	95.7 (3)	N1-Co1-O2	93.56 (18)	O24-La1-O29	65.99 (13)
N1-Co1-O8	92.5 (3)	N2-Co1-O2	178.11 (18)	O14-La1-O29	110.64 (13)
N2-Co1-O2	95.5 (3)	N1-Co1-O1	177.3 (2)	O24-La1-O27	68.90(14)
N1-Co1-O2	178.4 (3)	N1-Co1-N2	84.7 (2)	O14-La1-O27	107.52 (12)
O8-Co1-O2	87.8 (3)	N1-Co1-O2	93.56 (18)	O29-La1-O27	71.89 (14)
N2-Co1-O1	178.0 (3)	N2-Co1-O2	178.11 (18)	O24-La1-O23	48.55 (14)
N1-Co1-O1	94.0 (3)	N1-Co1-O1	177.3 (2)	O14-La1-O23	133.13 (14)
O8-Co1-O1	86.4(3)	N1-Co1-N2	84.7 (2)	O29-La1-O23	110.73 (14)
O2-Co1-O1	84.4 (3)	N1-Co1-O2	93.56 (18)	O27-La1-O23	66.46 (13)
N2-Co1-O4	87.7 (3)	N2-Co1-O2	178.11 (18)	O24-La1-O26	110.94 (14)
N1-Co1-O4	88.1 (3)	N1-Co1-O1	177.3 (2)	O14-La1-O26	64.72 (12)
O8-Co1-O4	176.6 (3)	N1-Co1-N2	84.7 (2)	O29-La1-O26	70.08 (13)
O2-Co1-O4	91.4 (3)	N1-Co1-O2	93.56 (18)	O27-La1-O26	47.96 (12)
O1-Co1-O4	90.3 (3)	N2-Co1-O2	178.11 (18)	O23-La1-O26	111.21 (13)
O10-Co2-O7	91.7 (3)	N7-Co2-N6	84.88 (19)	O24-La1-O21	71.91 (13)
O10-Co2-O3	88.4 (3)	N7-Co2-O6	177.20 (18)	O14-La1-O21	110.10 (12)
O7-Co2-O3	173.3 (3)	N6-Co2-O6	94.15 (17)	O29-La1-O21	70.17 (14)
O10-Co2-O1	156.9 (3)	N7-Co2-O5	93.15 (17)	O27-La1-O21	133.63 (14)
O7-Co2-O1	91.6 (3)	N6-Co2-O5	177.96 (17)	O23-La1-O21	103.85 (13)
O3-Co2-O1	85.8 (3)	O6-Co2-O5	87.79 (15)	O26-La1-O21	133.96 (13)
O10-Co2-O2	127.0 (3)	N7-Co2-N8	91.12 (18)	O24-La1-O18	117.68 (14)

Bond valence sum (BVS) calculations were carried out to determine the oxidation states of the metal atoms in complexes 1 and 2, and the results are summarized in Table 3 (Ghosh et al., 2019a). The calculated BVS data indicate that the two Co atoms in complex **1** are in +2 and +4 oxidations state, whereas in complex **2**, both the Co atoms are in +4 oxidation state, and the one La is in +3 oxidation state.

**Table 3:** Bond valence sums^b^ for complexes 1 and 2^a^.

**Complex 1**		**Complex 2**		
**Co**_**1**^IV^_	**Co**_**2**^II^_	**Co**_**1**^IV^_	**Co**_**2**^IV^_	**La**^**III**^
4.13	1.97	4.43	4.14	3.34

a *The oxidation state of a particular atom is the nearest integer to the underlined value*.

b *The underlined value is the closest to the charge for which it was calculated*.

In the molecular geometry of complexes **1** and **2**, it is observed that in complex **1**, out of the two cobalt centers, the *o*-VED coordinated Co center is in +4 oxidation state, and the other cobalt center is in +2 oxidation state. Similarly, in complex **2**, both the Co centers are in +4 oxidation state and coordinated to two separate *o*-VED ligands. Thus, in these two complexes, it implies that the cobalt center coordinated to *o*-VED ligand is in +4 oxidation state otherwise, which is relatively unstable compared to Co in +2 and +3 oxidation state. So, here the *o*-VEDH_2_ ligand may be playing an important role in stabilizing the +4 oxidation state of cobalt.

**Figure 2:**
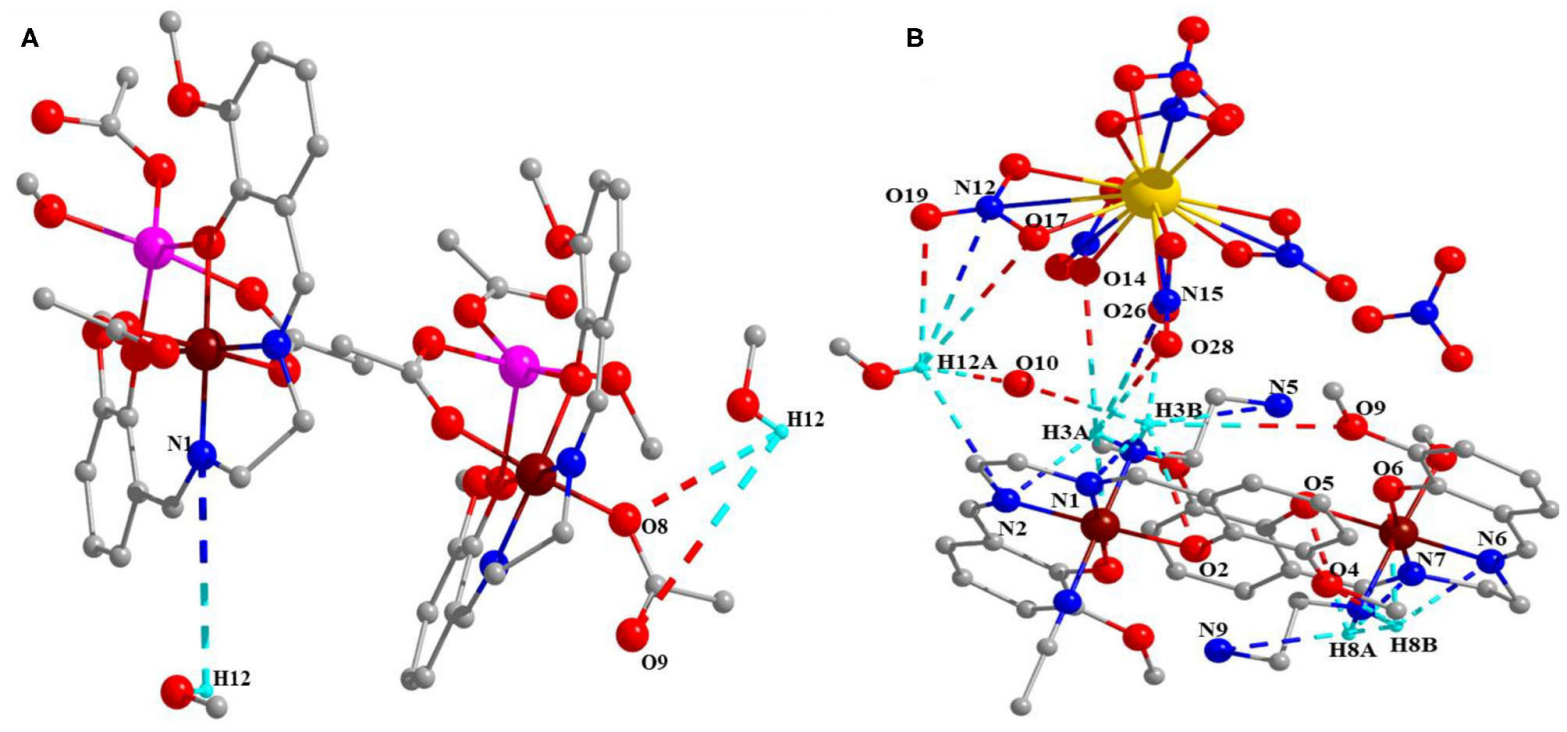
Intramolecular and intermolecular H-bonding **(A)** complex 1 and **(B)** complex 2.

**Figure 3:**
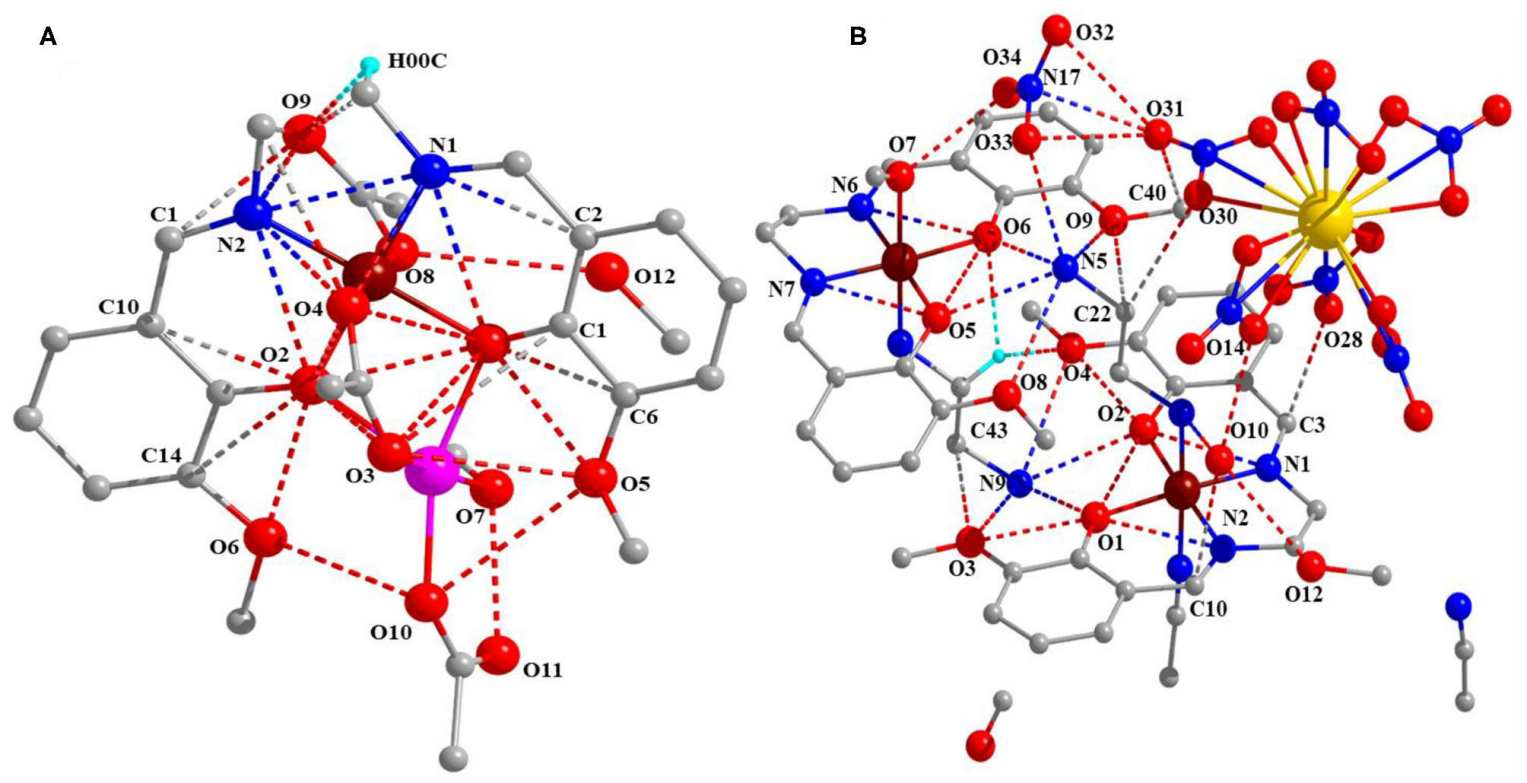
Van der Waals interaction **(A)** complex 1 and **(B)** complex 2.

Moreover, these complexes were stabilized through intramolecular and intermolecular H-bonding (Figure 2) and van der Waals interactions (Figures 3A,B), and the corresponding bond lengths are shown in Table 4 and ESI Supplementary Table 1, respectively.

**Table 4:** Selected H-bond length (Å) for complexes 1 and 2.

**Complex 1**		**Complex 2**	
**Bond type**	**Bond length (Å)**	**Bond type**	**Bond length (Å)**
O8 … H12	3.233	H12A … O17	3.867
H12 … O9	4.598	H12A … O19	3.392
N1 … H12	4.119	H3B … O26	2.501
		H3B … N5	3.942
		H3A … O25	3.999
		H3A … O26	3.202
		H3A … O28	3.9917

**Figure 4:**
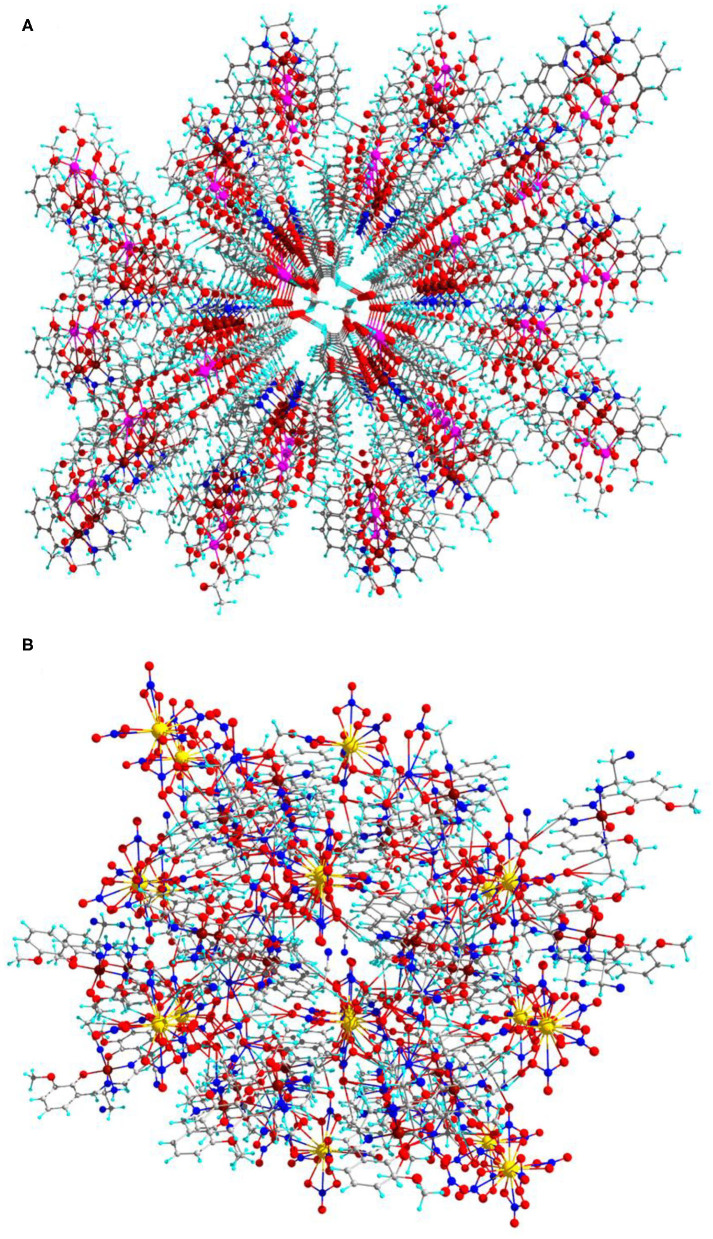
Catenation 2D layers MOF **(A)** for complex 1 and **(B)** for complex 2 (central projection, along 100-a axis) in ball-and-stick model.

Interestingly, complexes **1** and **2** have MOF structures those are constructed through catenation of 2D layers as shown in Figure 4. Among the two complexes, complex 1 has an excellent MOF channel with size side of 2.44 ×6.221 Å along with diagonal side of 5.88 ×7.601 Å as shown in ESI Supplementary Figure 1.

### Fourier Transform Infrared Spectroscopy (FTIR) Analysis

**Figure 5:**
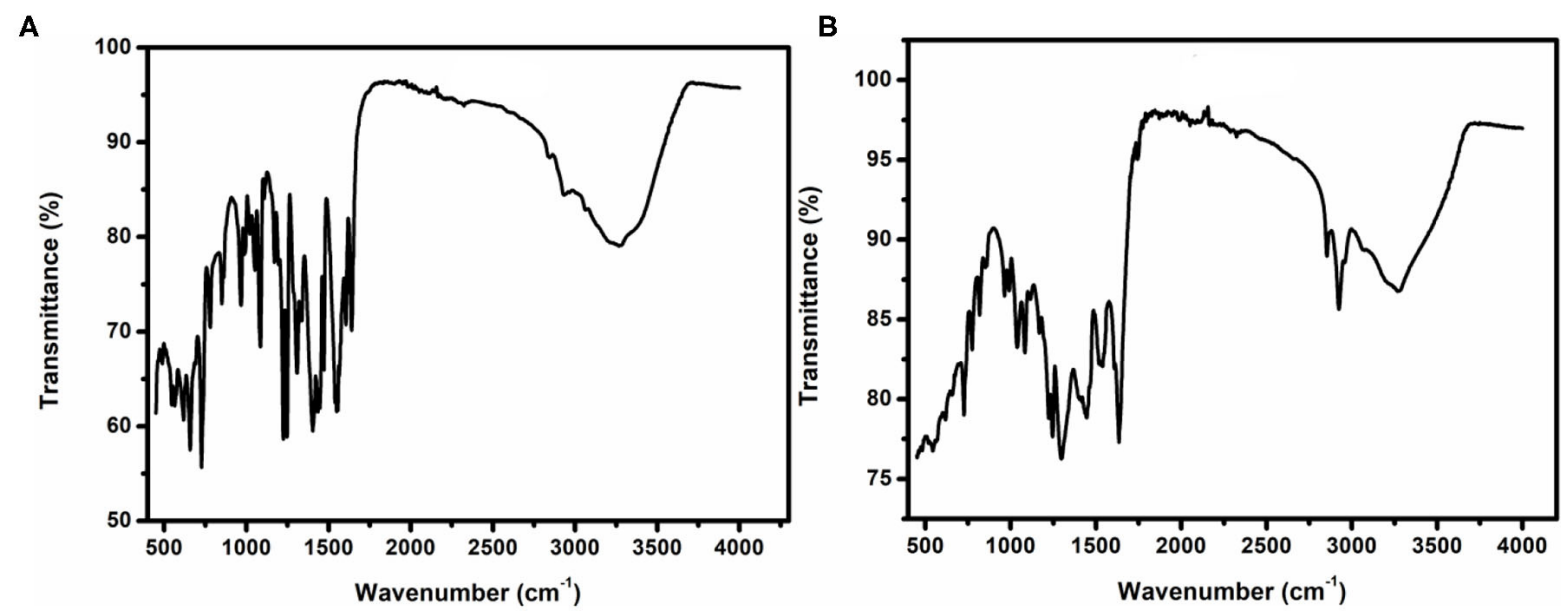
FT-IR of **(A)** complex 1 and **(B)** complex 2.

The FTIR spectra of complexes **1** and **2** are displayed in Figure 5, which shows the major peaks, wavenumber, and the interpretation of the possible functional groups. Selected IR data for complex **1** in cm^−1^: 488 (b) for Co-N, 544 (m) for Co-O, 1,082 (m) For C-O, 1,225 (s) for C-N, 1,555 (m) for C=N, 1,638 (m) for C=C, and 3,260 (b) for C-H.

Selected IR data for complex **2** in cm^−1^: 485 (b) for Co-N, 501 (b) for La-O, 545 (b) for Co-O, 1,082 (m) For C-O, 1,246 (s) for C-N, 1,607 (b) for C=N, 1,634 (m) for C=C, and 3,268 (b) for C-H.

### Magnetic Study

#### DC Magnetic Study

**Figure 6:**
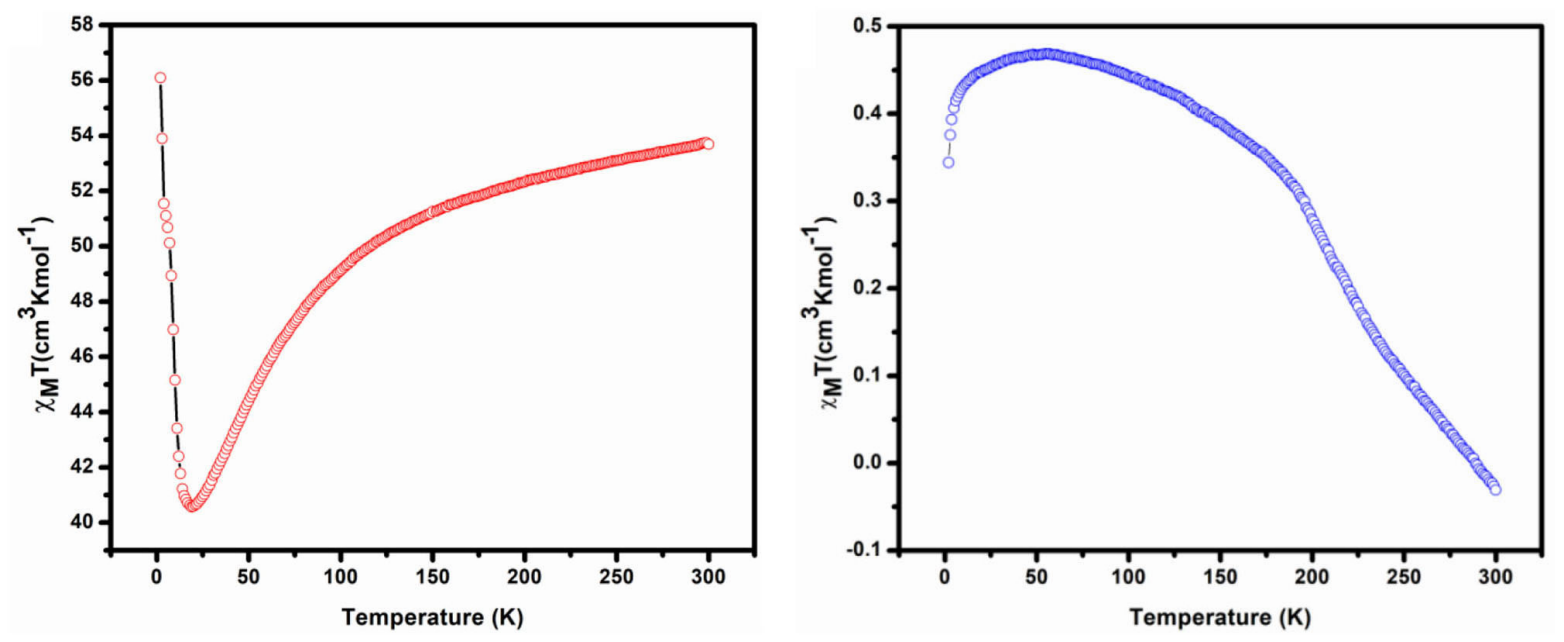
DC magnetic susceptibility plot of χ_M_T vs. T for complex 1 **(Left)** 2 **(Right)**.

The DC magnetic susceptibility of complexes (restrained in eicosane to prevent torqueing) was investigated under 3–300 K temperature and 0.1 T applied field. Figure 6 shows the plot of χ_M_T vs. T for complexes **1** and **2**. The observed room temperature value of χ_M_T for compound **1** is 53.7 cm^3^ mol^−1^ K. This value is the sum of contributions from both the Co^II^ and Co^IV^ in an uncoupled regime. We can calculate the expected contribution to the room temperature momentum from the Co^II^ ion (13.1 cm^3^ mol^−1^ K) using the following value Co^II^ (^4^F_9/2_, *S* = 3/2, *L* = 0, *g* = 2). However, the calculation of the contribution from the Co^IV^ ion is not as straightforward. The χ_M_T value appears to be higher than those expected in the spin-only case: Co^IV^ (^6^S_5/2_, *S* = 5/2, *L* = 0, *g* = 2). Upon cooling down to 19 K, it steadily decreases to a value of 40.6 cm^3^ mol^−1^ K and down to 2 K, after it increases dramatically up to 56.1 cm^3^ mol^−1^ K, which suggests either ferromagnetic interaction or ferromagnetic behavior.

The χ_M_T value in room temperature of complex **2** is −0.03 cm^3^ mol^−1^ K, which is very low than expected value of non-interacting two Co^IV^ ions (^6^S_5/2_, *S* = 5/2, *L* = 0, *g* = 2) and one La^III^ (^1^S_0_, *S* = 0, *L* = 0, *g* = 1). The χ_M_T for complex **2** remains constant at a value of approximately −0.03 cm^3^ mol^−1^ K at 300 K, down to 55 K; it steadily increases to a value 0.47 cm^3^ mol^−1^ K and down to 2 K at 0.34 cm^3^ mol^−1^ K with dramatic decrease, which suggests either antiferromagnetic interaction or antiferromagnetic behavior. Total spin (*S*) and corresponding exchange coupling constant (*J*) values for complexes **1** and **2** are 4 and 5 and 0.260 cm^−1^ and 0.043 cm^−1^ respectively.

#### AC Magnetic Study

**Figure 7:**
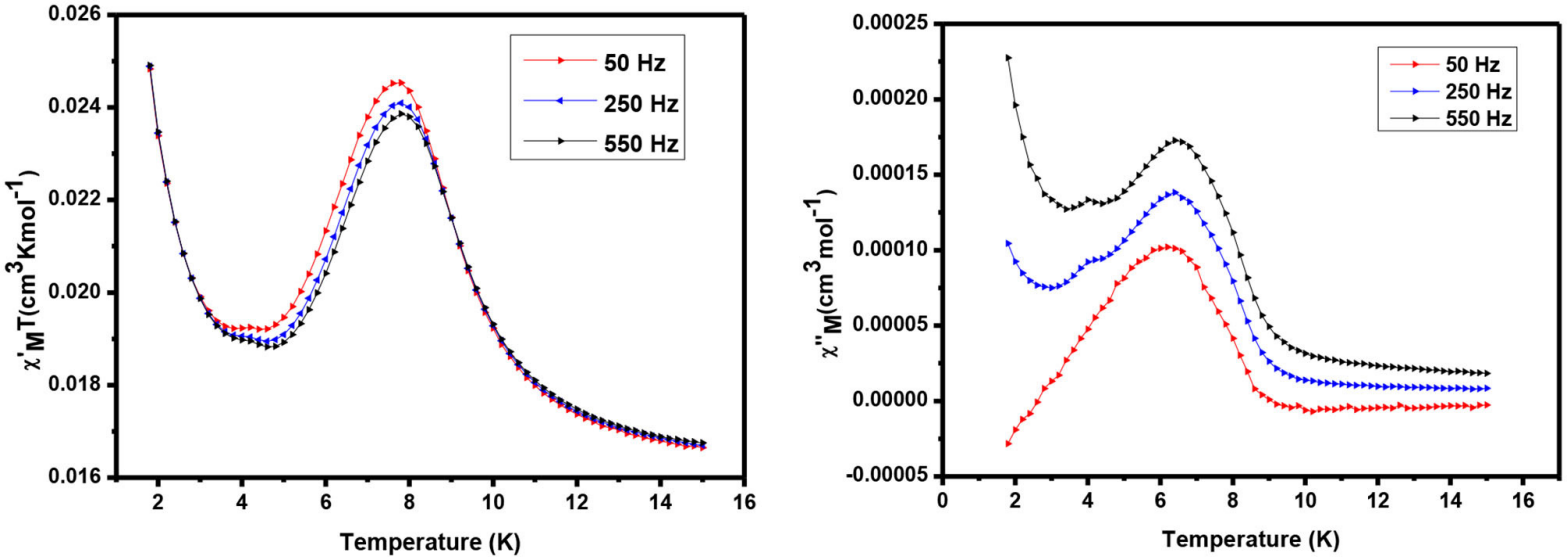
AC susceptibility of complex 1 in phase **(Left)** and out of phase **(Right)**.

**Figure 8:**
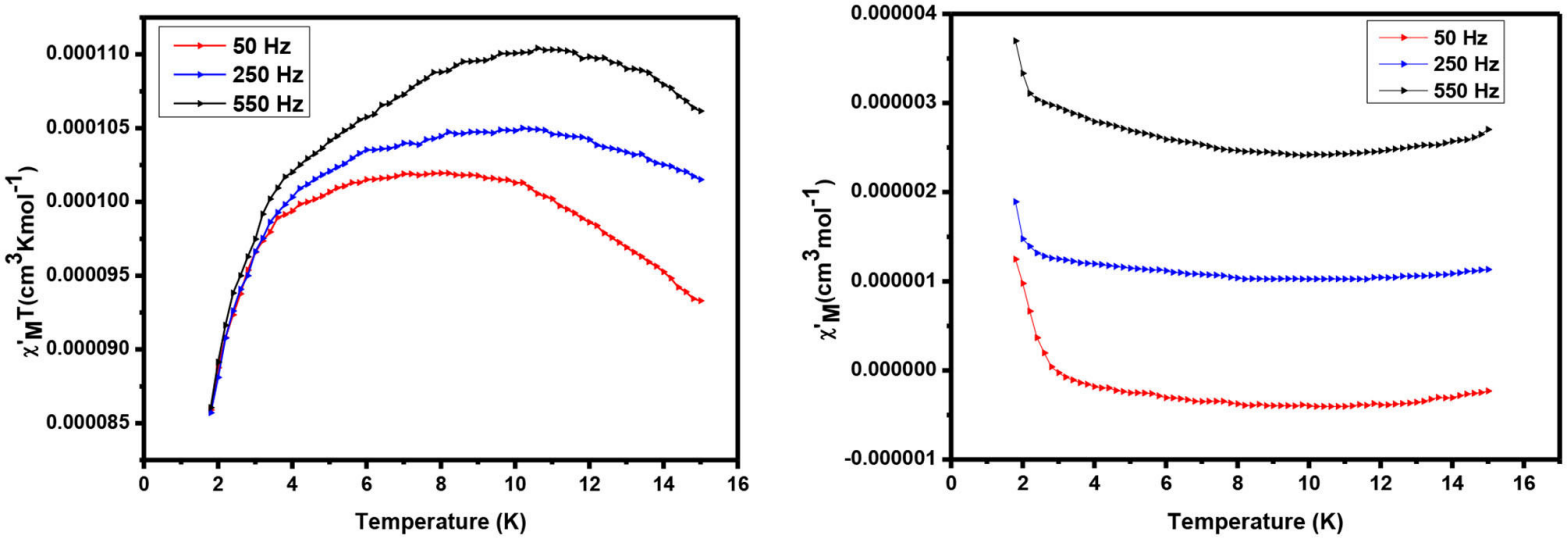
AC susceptibility of complex 2: in phase **(Left)** and out of phase **(Right)**.

AC susceptibility (in phase and out phase) studies have been conducted for the complexes in between 1.8 and 15 K in a zero applied field with 3.5 Oe driving field to investigate for slow magnetic relaxation, i.e., SMM behavior. The AC susceptibility studies for complexes **1** and **2** have been performed at various frequencies such as 50, 250, and 550 H, and a plot of χ_M_T vs. temperature is presented in Figures 7, 8, respectively.

The alternating current (ac) in-phase susceptibility of complexes **1** and **2** is in good agreement with the direct current (dc) data at the same temperature. In the case of complex **1**, χ_M_T value is a sharp decrease from 2 to 5 K, and then it is dramatically increased from 5 to 8 K, reaching the maximum $\chi_{\text{M}}\prime \text{T}$ value of 0.025 cm^3^ Kmol^−1^ (Figure 7) at about 7 K temperature. The frequency-dependent rise in the out-of-phase susceptibility is observed as a peak tail, indicating that **1** displays behavior characteristic of a SMM.

For complex **2**, $\chi_{\text{M}}\prime \text{T}$ value is significantly increased with increasing the temperature, having a maximum value of 1.104 cm^3^ Kmol^−1^ at 10 K. Similarly, **1** continues to show a decrease in $\chi_{\text{M}}\prime \text{T}$ with increasing temperatures before a slight frequency-dependent drop is observed at low temperature. Also equal to **1**, a frequency-dependent rise in the out-of-phase susceptibility is observed as peak tail, indicating that **2** also displays behavior characteristic of a SMM.

### Other Studies

#### Optical Properties

The optical band gap of the complexes has been computed from Tauc's equation (**1)** to explain the conductivity of isolated complexes:

(1) $${\left(\alpha h\upsilon \right)}^{n}=A\;\left(h\upsilon -E_{g}\right)$$

where α denotes the absorption coefficient, and it is calculated by α = (2.303 × absorption)/*t, t* = cubet thickness (1 cm), *A* refers to constant, *E*_*g*_ indicates the band gap energy, exponent *n* depends on the type of transition, and *h* denotes the Planck's constant.

**Figure 9:**
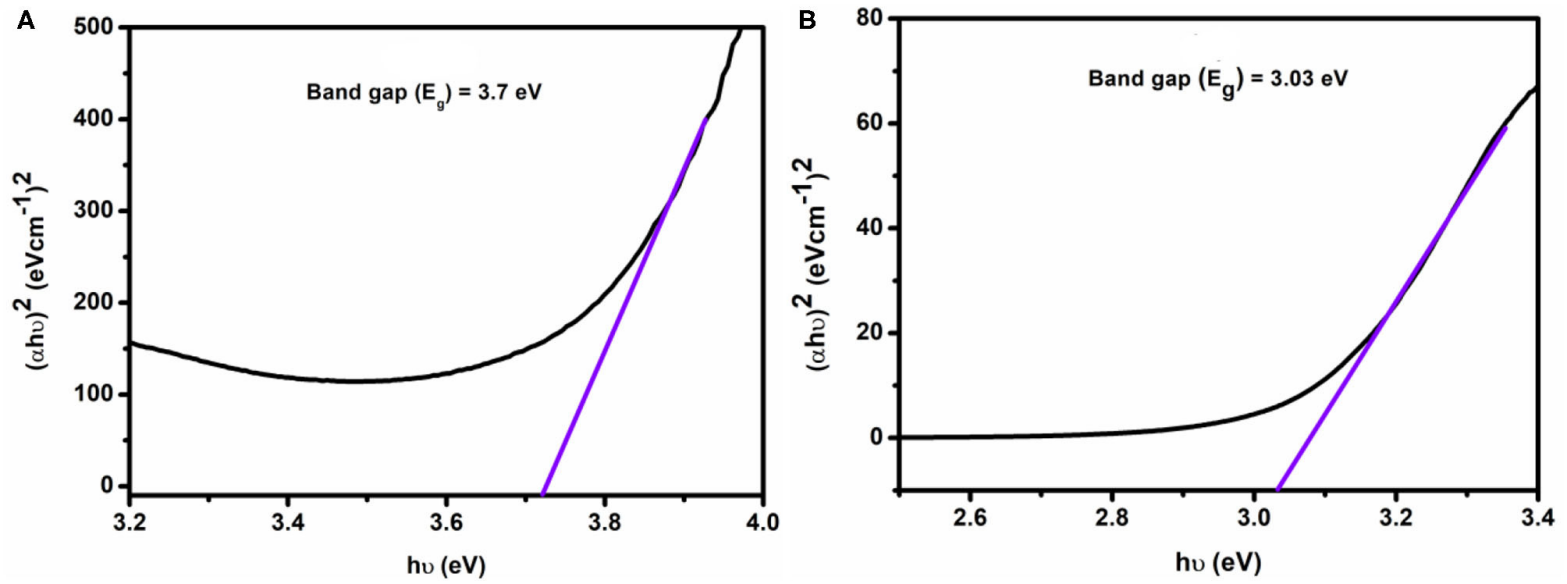
Band gap energy of complexes 1 **(A)** and 2 **(B)**.

Equation **1** was used to plot (α*h*υ)^2^ vs. *h*υ (Figure 9) from which band gap energy (*E*_*g*_) of the complexes was calculated by extrapolating the linear portion of the curve to (α*h*υ)^2^ = 0. The values of band gap energy of complexes **1** and **2** were 3.7 and 3.03 eV, respectively. These results are significant for solar, optoelectronic, and electronic applications because of a small band gap in energy, which extends the electronic progress from HOMO-LUMO to the increased electrical conductivity of the complexes (Brown and Altermatt, 1985; Sengupta et al., 1998; Turan et al., 2014; Ahmed et al., 2016). The band gap value suggests that complexes may act as semiconductor materials.

#### H_2_O_2_ Sensing

**Figure 10:**
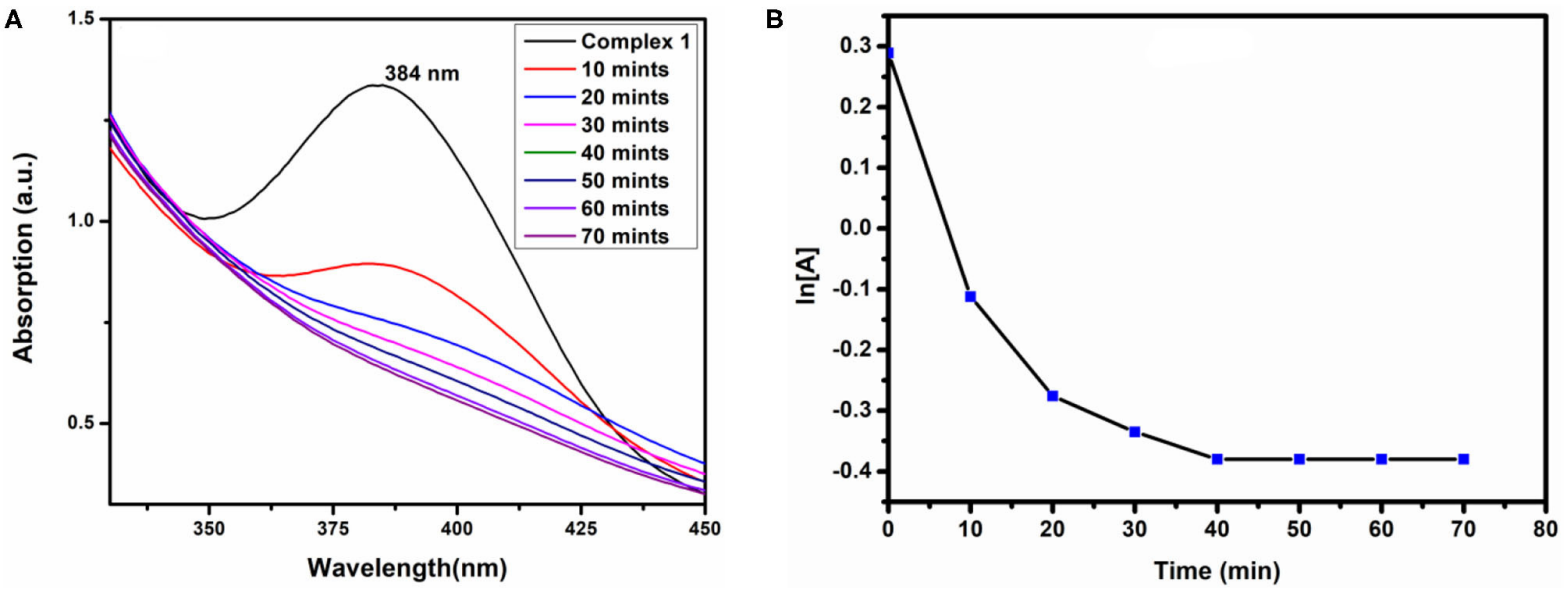
H_2_O_2_ sensing **(A)** and first order of reaction **(B)** of complex 1.

To determine the sensing capacity of the complexes we have used H_2_O_2_ as source materials. We have added 1 mL of 20 mM H_2_O_2_ to 3 mL solution of the complexes, and the electronic spectra were recorded at regular intervals. Figure 10A shows that the absorbance of complex **1** decreased with increasing the time in presence H_2_O_2_. After 10 min, we have observed that the characteristic peak of complex **1** at 274 nm was disappearing, and the color intensity of complex **1** turns from faded yellow to colorless. This is possible for the presence of Co(II), which should be oxidized to Co(III), and OH could be formed. During the decomposition reactions of H_2_O_2_ by complex **1**, hydroxyl radicals (OH) are probably formed and acted as an oxidant, which oxidizes Co(II) to Co(III)/Co(IV) and itself produces H_2_O; as a result, the absorbance of the solution mixture was decreased as observed in the UV-visible spectra. But such kind of H_2_O_2_ decomposition was not observed in complex **2**, which may be because Co sites already exist in higher (+4) oxidation state, and they do not form radicals. Therefore, complex **2** does not show the H_2_O_2_ decomposition properties. The degradation of complex **1** was followed by first-order kinetic reaction, and the rate constant (*k*) was found to be 1.59 × 10^−4^ S^−1^ shown in Figure 10B.

#### DNA Binding

**Figure 11:**
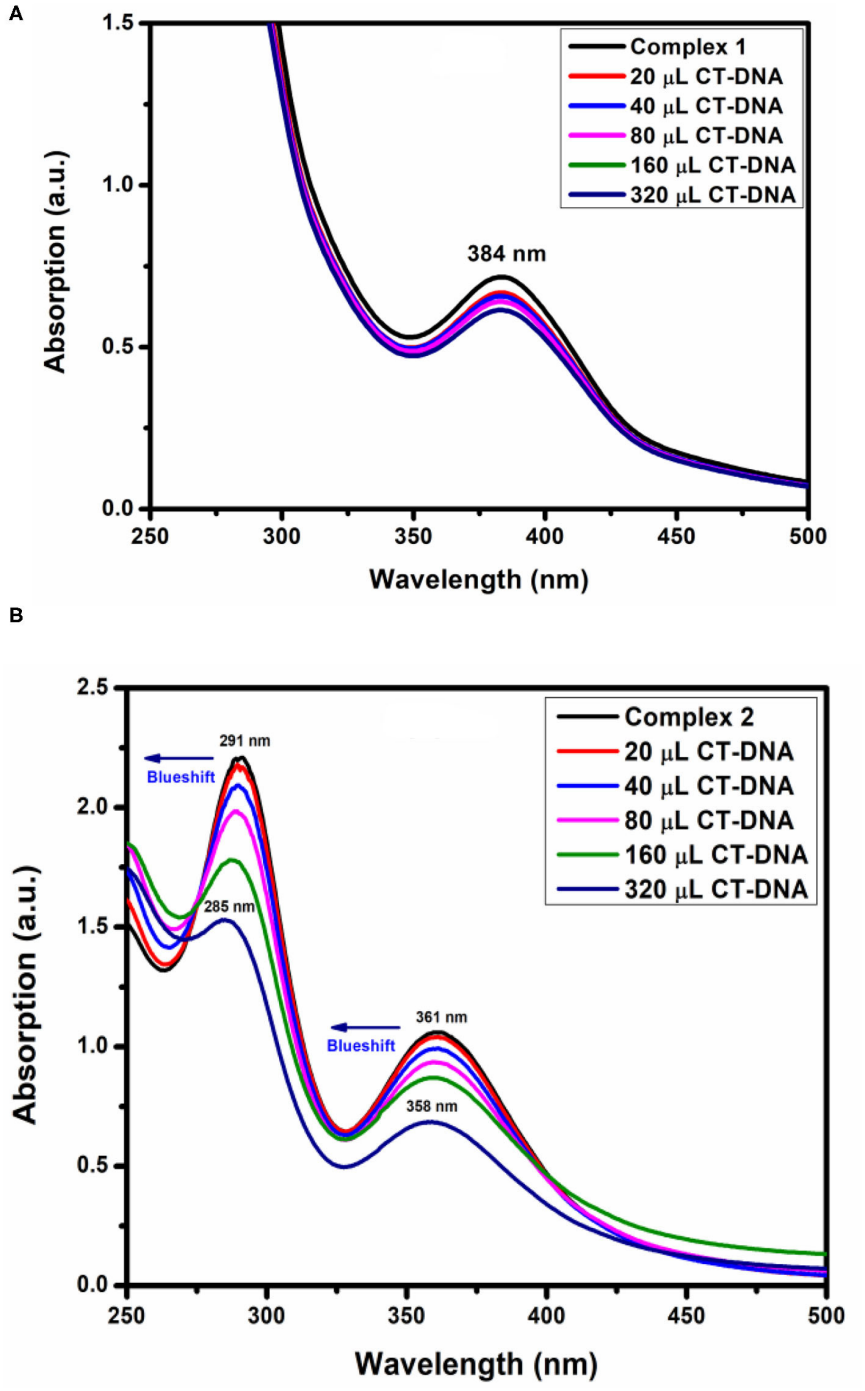
DNA-binding study **(A)** complex 1 and **(B)** complex 2.

Before adding CT-DNA to the complexes, the stability of complex **1**, complex **2**, and *o*-VEDH_2_ in Tris-HCl buffer solution was measured by UV-visible spectroscopy at room temperature for 1 h (each in 150-min interval), and the absorption peaks were not changed. The UV-visible absorption spectra of complexes **1** and **2** are represented in Figures 11A,B, respectively. The absorption peaks at 384 nm of complex **1** and at 291 and 361 nm of complex **2** are assigned for the d–d transition. To study the binding characteristics of complexes **1** and **2** with CT-DNA, CT-DNA was stepwise added to the solution of the complexes in a cuvette separately, and absorption spectra were recorded in each step. In case of complex **1**, except slight decrease in absorption, no characteristic change was observed. However, in case of complex **2** upon stepwise addition of 20, 40, 60, and 80 μL of CT-DNA, the characteristic absorption at 291 gradually shifts to 289, 288, 286, and 285 nm (blue shift), whereas the characteristic peak at 361 nm gradually changes to 360, 359, and 358 nm (blue shift), respectively. This change in absorption characteristics indicates some short of interaction between complex **2** and CT-DNA; however, the nature of this interaction is yet to be established. Complex 2 contains nitrate, which could form H-bond with CT-DNA shown in ESI S2, whereas for complex **1**, the capability to form H-bond with CT-DNA is probably lower because all O, N, and H atoms of complex 1 should be busy in formation of intramolecular H-bonding.

The absorption spectra of the complex binding to DNA may be through intercalation, revealing that blue shift appears because of strong staking interaction between complex **2** and base pairs of DNA. The binding constant *K*_*b*_ of complex **2** was determined by the equation (Fu et al., 2005) as follows:

$$\begin{array}{l} \left[DNA\right]\times {\left(\varepsilon_{a}-\varepsilon_{f}\right)}^{-1}=\left[DNA\right]\times {\left(\varepsilon_{b}-\varepsilon_{f}\right)}^{-1}+K_{b}^{\;-\text{1}}\\ \;\times {\left(\varepsilon_{b}-\varepsilon_{f}\right)}^{-1} \end{array}$$

where **ε**_***a***_, **ε**_***f***_, and **ε**_***b***_ were denoted as extinction coefficient of the complex, CT-DNA, and bound complex, respectively. The binding constant of complex **2** was 1.22 × 10^5^ M^−1^ shown in Table 5. Therefore, binding constant values lie within the range of a characteristic of the CT-DNA binding by complexes through intercalative mode (Wolfe and Shimer, 1987; Patel et al., 2011).

**Table 5:** Absorption titration data of complexes **1** and **2**.

**Complex 2**				
**Absorption**	**[DNA]/(ε*_***a***_* – ε*_***f***_*) × 10^**7**^ M^**2**^ cm**	**[DNA] × 10^**4**^ M**	***K_***b***_* (M^**−1**^)**	
1.063	0.000	0.000	1.217 × 10^5^ M^−1^	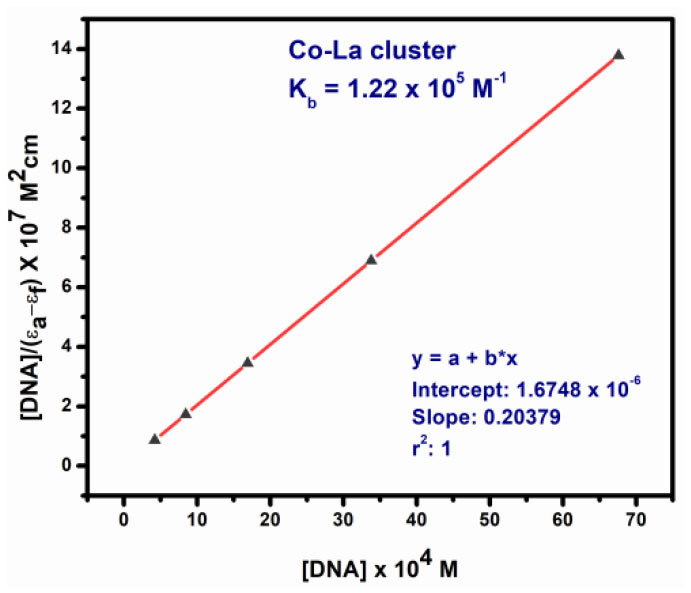
1.038	0.861	4.226		
0.988	1.722	8.451		
0.937	3.446	16.909		
0.870	6.889	33.806		
0.686	13.779	67.611		

## Conclusion

In the present work, we have successfully synthesized two novel Co^II^Co^IV^ and ${\text{Co}}_{2}^{\text{IV}}$La^III^ complexes utilizing *o*-VEDH_2_ as Schiff base ligand from two different reaction conditions and investigated their SMMs properties, as well as multifunction activities, such as H_2_O_2_ sensors and DNA binders. In solid state, both the complexes show the formation of MOFs structures. Complexes **1** and **2** have optical band gap energy of 3.7 and 3.03 eV, which indicates that these may have semiconductor properties. Interestingly, it is also established that complex **1** has H_2_O_2_-sensing properties with a rate constant (*k*) = 1.59 × 10^−4^ s^−1^, whereas complex **2** is bound to CT-DNA by intercalation binding mode with binding constant *k*_b_ = 1.22 × 10^5^ M^−1^.

## Experimental

### Materials and Methods

All the reagent-grade chemicals were purchased from Sigma–Aldrich, India, and were used as received without further purification. All the reaction solvents were also used as received.

### Syntheses

#### Schiff Base Ligand

**Scheme 1:**
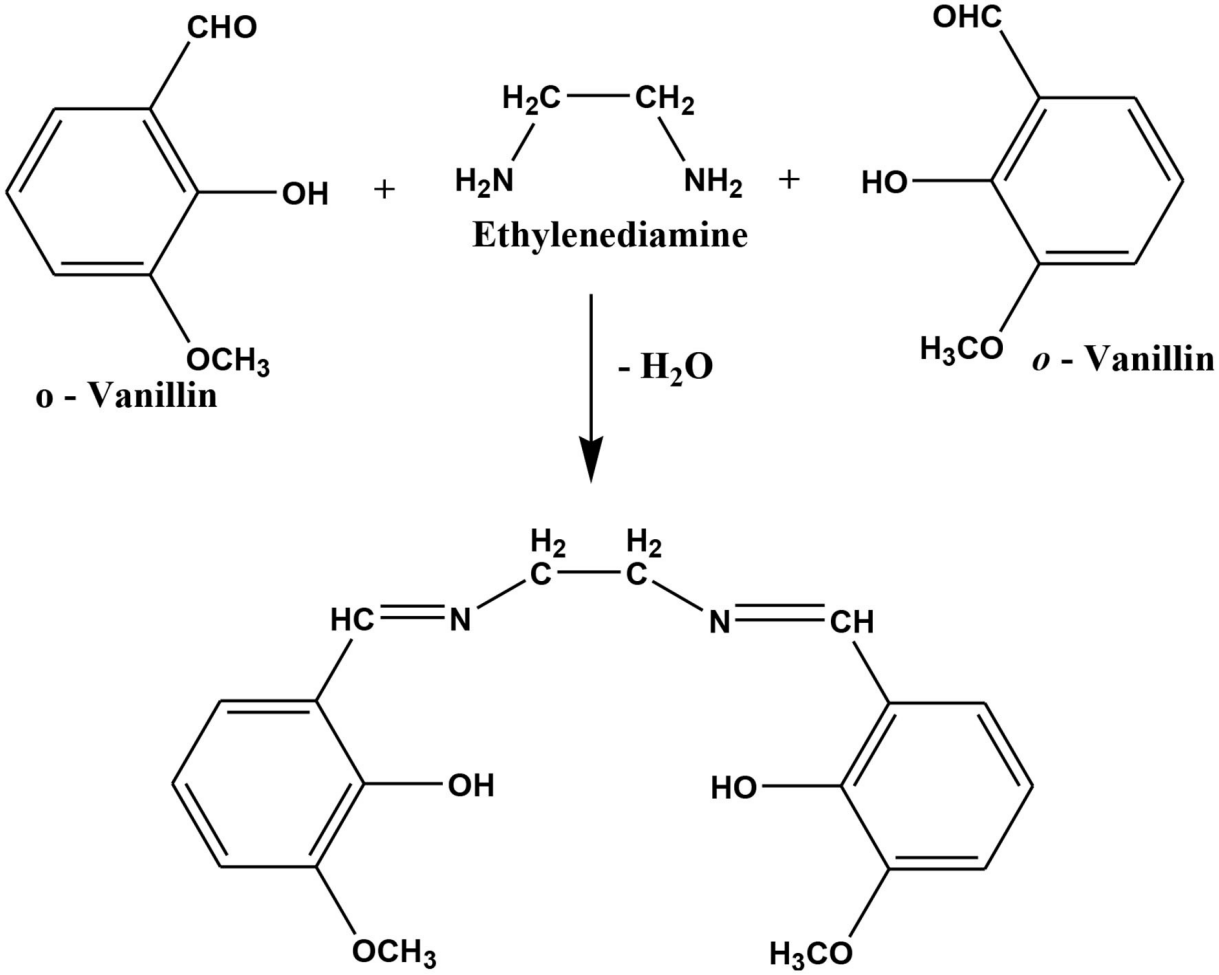
Preparation of Schiff base ligand *N,N*′-bis(*o*-vanillinidene)ethylenediamine (*o*-VEDH_2_) (Ghosh et al., 2019b).

The Schiff base ligand N,N′-bis(*o*-vanillinidene) ethylenediamine (*o*-VEDH_2_) was synthesized in the following procedure (Scheme 1) and also reported in our earlier publication (Ghosh et al., 2019b):

(1) $$[Co^{\text{II}}Co^{\text{IV}}(o-VED){\left(OAc\right)}_{2}(\cdot \mu_{2}-OAc)(OMe)]•MeOH$$

Co(acac)_2_ (0.487g, 3 mmol) was dissolved in 10 mL methanol followed by dropwise addition of *o*-VEDH_2_ (0.305g, 1 mmol) (separate solution in MeCN) and stirred for more than 2 h until a clear solution was obtained. Finally, the resulting solution was filtered and divided into two parts. One part of the black color filtrate was kept for slow evaporation, and another part was layered over diethyl ether. In a week, black single crystals were obtained in both the conditions in high yield. The single crystals were separated out from the mother solution and were washed with diethyl ether (two to three times). The analytically pure compound was obtained after drying under vacuum.

Yield: 33% (60 mg). Anal. Calcd. for **C**_**26**_ **H**_**34**_**Co**_**2**_**N**_**2**_**O**_**12**_ **(1)**: C, 28.32; H, 5.12; N, 3.94%. Found: C, 27.91; H, 4.89; N, 3.88%. Selected IR data in cm^−1^: 488 (b), 544 (m), 1,082 (m), 1225 (s), 1,555 (m), 1,638 (m), and 3,260 (b) (s = strong, m = medium, b = broad). Detailed discussion of IR spectrum of **1** is provided in Figure 5A.

(2) $$\begin{array}{l} \left[Co_{2}^{\text{IV}}{\left(o-VED\right)}_{2}{\left(en\right)}_{2}\left(NCCH_{3}\right)\left(OCH_{3}\right)\right]\\ {}[La{\left(NO_{3}\right)}_{6}]\left(NO_{3}^{-}\right)•2MeOH•MeCN•H_{2}O \end{array}$$

Solution of La(NO_3_)_3_·6H_2_O (0.054g, 1 mmol) in MeOH (10 mL) was added dropwise to a stirred solution of *o*-VEDH_2_ (0.081g, 2 mmol) in MeCN (10 mL). The reaction mixture was kept stirring, and solid Co(NO_3_)_2_·6H_2_O (0.037g, 1 mmol) was added after 15 min. Further, solid iodine (0.063 g, 2 mmol) was added to the reaction mixture and was vigorously stirred for additional 2 h to obtain a black color solution. The black color solution was filtered, and the filtrate was divided into two parts. One part of the filtrate was kept for slow evaporation, and the other part was layered over CH_3_Cl. In a week, high-quality black single crystals were obtained from the CH_3_Cl layered solution in high yield. The crystals were separated out and were washed with diethyl ether (two to three times) before drying under vacuum.

Yield: 20% (1.2 mg). Anal. Calcd. for **C**_**47**_**H**_**58**_**Co**_**2**_**LaN**_**17**_**O**_**33**_ **(2)**: C, 28.32; H, 3.55; N, 14.37%. Found: C, 28.34; H, 3.52; N, 14.29%. Selected IR data in cm^−1^: 485 (b), 501 (b), 545 (b), 1,082 (m), 1,246 (s), 1,607 (b), 1,634 (m), and 3,268 (b) [s = strong, m = medium, b = broad]. Detailed discussion of IR spectrum of **2** is provided in Figure 5B.

#### Physical Measurements

The ^1^H NMR spectra of Schiff base ligand were recorded in a JEOL 400-MHz spectrophotometer. Infrared spectra were recorded in a PerkinElmer FTIR spectrometer in the solid state (KBr pellets) in the 400- to 4,000-cm^−1^ range. Elemental analyses (C, H, and N) and X-ray crystallography were performed through central facilities at IACS, Kolkata. Shimadzu UV-1800 spectrophotometer was used to record the electronic spectrum of complexes. Quantum Design MPMS-XL SQUID magnetometer (IISER Bhopal) equipped with a 7-T magnet and operating in the 1.8- to 300-K range was used on vacuum dried solids to collect variable-temperature dc and ac magnetic susceptibility data. Samples were embedded in solid eicosane in a gel capsule to prevent torqueing. Diamagnetic and background contributions from the eicosane and gel capsule were measured as a blank and subtracted from the susceptibility. Magnetization vs. field and temperature data was fit using the program MAGNET (Davidson, 1999). Pascal's constants (Weast and Astle, 1984) were used to estimate the diamagnetic correction, which was subtracted from the experimental susceptibility to give the molar paramagnetic susceptibility (**χ**_M_).

### Crystallographic Data Collection and Refinement

On the Bruker SMART APEX CCD diffractometer with SMART/SANTI programming, a single-precious-stone X-beam data were collected for complexes **1** and **2** at 293 K/100 K (Sheldrick, 2008). MoKα graphite monochromatized (0.71073 Å) radiation was used for the collection of intensity information at 100 K. A coordinated strategy using SHELXS-2013, which was incorporated into WinGX, understood the structures (Farrugia, 2012, 2013; Sheldrick, 2013) SADABS was associated with exact corrections to the absorption (Sheldrick, 1996). An anisotropic displacement coefficient was used to refine all non-hydrogen particles. The particles of hydrogen carbon were integrated into geometric positions and given warm parameters 1, 2 those of the molecule to which they were attached. An initial search for reciprocal space exposed that both complexes are crystallized in monoclinic phase with P21/c and P21/nspace group.

The consequences of the precious crystal data and structure refined information of complexes **1** and **2** are available in Table 1, respectively.

### Hydrogen Peroxide Sensing Capacity of the Complexes

The H_2_O_2_ detection protocol is already discussed in our previous article (Bera and Raj, 2013; Ghosh et al., 2019b). Initially, complexes were dissolved in CH_3_CN to obtain a suitable dilute solution for the initial of UV-visible spectrum. In 3 mL of the above solution, 1 mL 20 mM H_2_O_2_ was added. Spectra were recorded at regular intervals in a UV-visible spectrophotometer.

### DNA-Binding Study

CT-DNA was dissolved in Tris-HCl buffer (pH 7.25) solution. and its purity was confirmed from the obtained absorbance ratio of A260/A280 in the range of 1.8 to 1.9. Metal complexes **1** and **2** were dissolved, and absorption titration experiments were performed using the increased concentration of CT-DNA with the constant amount of the metal complex.

## Data Availability Statement

The datasets presented in this study can be found in online repositories. The names of the repository/repositories and accession number(s) can be found in the article/Supplementary Material.

## Author Contributions

Synthesis and applications were performed by MKG. The crystallography of the complexes was performed by BJ. The manuscript was designed and written by MKG and TKG. All the authors have given approval to the final version of the manuscript.

## Conflict of Interest

The authors declare that the research was conducted in the absence of any commercial or financial relationships that could be construed as a potential conflict of interest.

## References

[B1] Ahmed A. H. Hassan A. M. Gumaa H. A. Mohamed B. H. Eraky A. M. Omran A. A. (2016). Copper (II)-oxaloyldihydrazone complexes: physico-chemical studies: energy band gap and inhibition evaluation of free oxaloyldihydrazones toward the corrosion of copper metal in acidic medium. Arab. J. Chem. 12, 4287–4302. 10.1016/j.arabjc.2016.05.015

[B2] Alghool S. El-Halim H. F. A. El-Sadek M. A. Yahia I. S. Wahab L. A. (2013). Synthesis, thermal characterization, antimicrobial activity of lanthanum, cerium, thorium complexes of amino acid Schiff base ligand. J. Ther. Anal. Calorimetry 112, 671–681. 10.1007/s10973-012-2628-4

[B3] Bera R. K. Raj C. R. (2013). A facile photochemical route for the synthesis of triangular Ag nanoplates and colorimetric sensing of H_2_O_2_. J. Photochem. Photobiol A: Chem. 270, 1–6. 10.1016/j.jphotochem.2013.07.005

[B4] Bogani L. Wernsdorfer W. (2010). “Molecular spintronics using single-molecule magnets,” *in Nanoscience and Technology: A Collection of Reviews from Nature Journals* (World Scientific), 194–201. 10.1142/9789814287005_0020 18297126

[B5] Brown I. D. Altermatt D. (1985). Bond-valence parameters obtained from a systematic analysis of the inorganic crystal structure database. Acta.Crystallogr. Sect. B 41, 244–247. 10.1107/S0108768185002063

[B6] Chelebaeva E. Larionova J. Guari Y. Ferreira R. A. Carlos L. D. Paz F. A. A.. (2009). Luminescent and magnetic cyano-bridged coordination polymers containing 4d– 4f ions: toward multifunctional materials. Inorg. Chem. 48, 5983–5995. 10.1021/ic900378d 20507102

[B7] Clough A. J. Skelton J. M. Downes C. A. de la Rosa A. A. Yoo J. W. Aron W.. (2017). Metallic conductivity in a two-dimensional cobalt dithiolene metal–organic framework. J. Am. Chem. Soc. 139, 10863–10867. 10.1021/jacs.7b05742 28704606

[B8] Davidson E. R. (1999). Indiana University: Bloomington, IN.

[B9] Ding Y. S. Chilton N. F. Winpenny R. E. Zheng Y. Z. (2016). On approaching the limit of molecular magnetic anisotropy: a near-perfect pentagonal bipyramidal dysprosium (III) single-molecule magnet. Angew. Chem. Int. Ed. 55, 16071–16074. 10.1002/anie.201609685 27874236

[B10] Dong Z. Sun Y. Chu J. Zhang X. Deng H. (2017). Multivariate metal–organic frameworks for dialing-in the binding and programming the release of drug molecules. J. Am. Chem. Soc. 139, 14209–14216. 10.1021/jacs.7b07392 28898070

[B11] Droge W. (2002). Free radicals in the physiological control of cell function. Physiol. Rev. 82, 47–95. 10.1152/physrev.00018.2001 11773609

[B12] Farrugia L. J. (2012). WinGX and ORTEP for Windows: an update. J. Appl. Crystallogra. 45, 849–854. 10.1107/S0021889812029111

[B13] Farrugia L. J. (2013). WinGX, version 3 2013, Department of Chemistry, University of Glasgow.

[B14] Freitas A. R. Rubira A. F. Muniz E. C. (2010). Polychloroprene degradation by photo-fenton. Conductivity measures as new approach for detecting/evaluation of degradation products. J. Polym. Environ. 18, 668–673. 10.1007/s10924-010-0226-8

[B15] Fu M. L. Guo G. C. Liu X. Liu B. Cai L. Z. Huang J. S. (2005). Syntheses, structures and properties of three selenoarsenatestemplated by transition metal complexes. Inorg. Chem. Commun. 8, 18–21. 10.1016/j.inoche.2004.10.021

[B16] Gao R.-C. Guo F.-S. Bai N.-N. Wu Y.-L. Yang F. Liang J.-Y.. (2016). Two 3D Isostructural Ln(III)-MOFs: displaying the slow magnetic relaxation and luminescence properties in detection of nitrobenzene and Cr_2_O_7^2-^_, *Inorg*. Chem. 55, 11323–11330. 10.1021/acs.inorgchem.6b01899 27759963

[B17] Gatteschi D. Sessoli R. (2003). Quantum tunneling of magnetization and related phenomena in molecular materials. Angew. Chem. Int. Ed. 42, 268–297. 10.1002/anie.200390099 12548682

[B18] Ghosh M. K. Chandraker S. K. Shukla R. Mandal M. Mandal V. Ghorai T. K. (2019a). Molecular interaction, antimicrobial, antioxidant, cytotoxic and magnetic properties of Mn12 benzoate. J. Cluster Sci. 31, 575–589. 10.1007/s10876-019-01633-5

[B19] Ghosh M. K. Pathak S. Ghorai T. K. (2019b). Synthesis of two mononuclear schiff base metal (M= Fe, Cu) complexes: MOF structure, dye degradation, H_0_O_2_ sensing, and DNA binding property. ACS Omega 4, 16068–16079. 10.1021/acsomega.9b02268 31592474 PMC6777120

[B20] Gupta S. K. Rajeshkumar T. Rajaraman G. Murugavel R. (2016). An air-stable Dy (III) single-ion magnet with high anisotropy barrier and blocking temperature. Chem. Sci. 7, 5181–5191. 10.1039/C6SC00279J 30155168 PMC6020529

[B21] Ishikawa N. Sugita M. Ishikawa T. Koshihara S. Y. Kaizu Y. (2003). Lanthanide double-decker complexes functioning as magnets at the single-molecular level. J. Am. Chem. Soc. 125, 8694–8695. 10.1021/ja029629n 12862442

[B22] King P. Wernsdorfer W. Abboud K. A. Christou G. (2005). Single-molecule magnets: a reductive aggregation route to new types of Mn12 complexes. Inorg.Chem. 44, 8659–8669. 10.1021/ic051150j 16296819

[B23] Koo Won-Tae. Choi Seon-Jin. Kim Sang-Joon. Jang Ji-Soo. Tuller H. L. Kim Il-Doo. (2016). Heterogeneous sensitization of metal–organic framework driven metal@metal oxide complex catalysts on an oxide nanofiber scaffold toward superior gas sensors, J. Am. Chem. Soc. 138, 13431–13437. 10.1021/jacs.6b09167 27643402

[B24] Kumar K. Stefańczyk O. Chorazy S. Nakabayashi K. Sieklucka B. Ohkoshi S.-I. (2019). Effect of noble metals on luminescence and single-molecule magnet behavior in the cyanido-bridged Ln–Ag and Ln–Au (Ln = Dy, Yb, Er) complexes. Inorg. Chem. 58, 5677–5687. 10.1021/acs.inorgchem.8b03634 30998322

[B25] Li H. G. Yang Z. Y. Wang B. D. Wu J. C. (2010). Synthesis, crystal structure, antioxidation and DNA-binding properties of the Ln complexes with 1-phenyl-3-methyl-5-hydroxypyrazole-4-carbaldhyde-(benzoyl) hydrazone. J. Organomet. Chem. 695, 415–422. 10.1016/j.jorganchem.2009.10.032

[B26] Long J. (2019). Luminescent schiff-base lanthanide single-molecule magnets: the association betweeen optical and magnetic properties. Front. Chem. 7:63 10.3389/fchem.2019.00063 30788341 PMC6373491

[B27] Lv J. Liu T. Cai S. Wang X. Liu L. Wang Y. (2006). Synthesis, structure and biological activity of cobalt (II) and copper (II) complexes of valine-derived schiff bases. J. Inorg. Biochem. 100, 1888–1896. 10.1016/j.jinorgbio.2006.07.014 16965817

[B28] Mandal A. Dasgupta S. Ganguly S. Bauzá A. Frontera A. Das D. (2017). Cooperative influence of pseudohalides and ligand backbone of Schiff-bases on nuclearity and stereochemistry of cobalt (III) complexes: experimental and theoretical investigation. Dalton Trans. 46, 15257–15268. 10.1039/C7DT03040A 29068013

[B29] Modak R. Sikdar Y. Thuijs A. E. Christou G. Goswami S. (2016). CoII4, CoII7, and a series of CoII2LnIII (LnIII= NdIII, SmIII, GdIII, TbIII, DyIII) coordination clusters: search for single molecule magnets. Inorg. Chem. 55, 10192–10202. 10.1021/acs.inorgchem.6b01402 27690397

[B30] Montgomery C. P. Murray B. S. New E. J. Pal R. Parker D. (2009). Cell-penetrating metal complex optical probes: targeted and responsive systems based on lanthanide luminescence. Acc. Chem. Res. 42, 925–937. 10.1021/ar800174z 19191558

[B31] Pages B. J. Ang D. L. Wright E. P. Aldrich-Wright J. R. (2015). Metal complex interactions with DNA. Dalton Trans. 44, 3505–3526. 10.1039/C4DT02700K 25427534

[B32] Patel M. N. Gandhi D. S. Parmar P. A. (2011). Synthesis, biological aspects and SOD mimic activity of square pyramidal copper (II) complexes with the 3rd generation quinolone drug sparfloxacin and phenanthroline derivatives. Inorg. Chem. Commun. 14, 128–132. 10.1016/j.inoche.2010.10.003

[B33] Pathak S. Ghosh M. K. Ghorai T. K. (2018). Luminescence, dye degradation and DNA binding properties of a dinuclear nona-coordinated Y (III) complex. Chem. Select. 3, 13501–13506. 10.1002/slct.201801826

[B34] Pathak S. Ghosh M. K. Mandal M. Mandal V. Bhattacharyya A. Ghorai T. K. (2019). Synthesis of a new acetate bridged Cu (ii) building block generated 1D polymer and studies on structural, magnetic, antibacterial and anticancer properties. New J. Chem. 43, 2019–2029. 10.1039/C8NJ04937H

[B35] Qi X. Tian H. Dang X. Fan Y. Zhanga Huimin Zhao (2019). A bimetallic Co/Mn metal–organic-framework with a synergistic catalytic effect as peroxidase for the colorimetric detection of H_2_O_2_. Anal. Methods 11, 1111–1124. 10.1039/C.8A.Y.02514B

[B36] Santini C. Pellei M. Gandin V. Porchia M. Tisato F. Marzano C. (2013). Advances in copper complexes as anticancer agents. Chem. Rev. 114, 815–862. 10.1021/cr400135x 24102434

[B37] Sengupta S. K. Pandey O. P. Srivastava B. K. Sharma V. K. (1998). Synthesis, structural and biochemical aspects of titanocene and zirconocene chelates of acetylferrocenylthiosemicarbazones. Transition Met. Chem. 23, 349–353. 10.1023/A:1006986131435

[B38] Sessoli R. Gatteschi D. Caneschi A. Novak M. A. (1993a). Magnetic bistability in a metal-ion cluster. Nature 365:141 10.1038/365141a0

[B39] Shahabadi N. Kashanian S. Darabi F. (2010). DNA binding and DNA cleavage studies of a water soluble cobalt (II) complex containing dinitrogen Schiff base ligand: the effect of metal on the mode of binding. Eur. J. Med. Chem. 45, 4239–4245. 10.1016/j.ejmech.2010.06.020 20598781

[B40] Sheldrick G. M. (1996). 2010 SADABS. Göttingen: University of Göttingen.

[B41] Sheldrick G. M. (2008). A short history of SHELX. ActaCrystallogr. Sec. A. 64,112–122. 10.1107/S0108767307043930 18156677

[B42] Sheldrick G. M. (2013). ShelXL2013. Germany: University of Göttingen.

[B43] Tang J. Zhang P. (2015). Lanthanide Single Molecule Magnets, Berlin: Springer 41-90. 10.1007/978-3-662-46999-6_2

[B44] Tasiopoulos A. J. Vinslava A. Wernsdorfer W. Abboud K. A. Christou G. (2004). Giant single-molecule magnets: a {Mn84} torus and its supramolecular nanotubes. Angew. Chem. Int. Ed. 43, 2117–2121. 10.1002/anie.200353352 15083460

[B45] Tredwin C. J. Naik S. Lewis N. J. Scully C. B. E. C. (2006). Hydrogen peroxide tooth-whitening (bleaching) products: review of adverse effects and safety issues. Br. Dent. J. 200, 371–376. 10.1038/sj.bdj.4813423 16607324

[B46] Turan N. Gündüz B. Körkoca H. Adigüzel R. Çolak N. Buldurun K. (2014). Study of structure and spectral characteristics of the Zinc (II) and Copper (II) complexes with 5, 5-Dimethyl-2-(2-(3-nitrophenyl) hydrazono) cyclohexane-1, 3-dione and their effects on optical properties and the developing of the energy band gap and investigation of antibacterial activity. J. Mex. Chem. Soc. 58, 65–75.

[B47] Van Slageren J. (2011). ;New directions in electron paramagnetic resonance spectroscopy on molecular nanomagnets, in EPR spectroscopy (Berlin: Springer) 199–234. 10.1007/128_2011_303 22076082

[B48] Vincent R. Klyatskaya S. Ruben M. Wernsdorfer W. Balestro F. (2012). Electronic read-out of a single nuclear spin using a molecular spin transistor. Nature 488:357. 10.1038/nature11341 22895342

[B49] Wang B. D. Yang Z. Y. Wang Q. Cai T. K. Crewdson P. (2006). Synthesis, characterization, cytotoxic activities, and DNA-binding properties of the La (III) complex with Naringenin Schiff-base. Bioorg. Med. Chem. 14, 1880–1888. 10.1016/j.bmc.2005.10.031 16310358

[B50] Wang J. Liu X. Sun Y. Yan L. He S. (2011). Synthesis, crystal structures, thermal properties, and DNA-binding studies of transition metal complexes with imidazole ligands. J. Coord. Chem. 64, 1554–1565. 10.1080/00958972.2011.575937

[B51] Wang Q. Huang Y. Zhang J. S. Yang X. B. (2014). Synthesis, characterization, DNA interaction, and antitumor activities of La (III) complex with Schiff base ligand derived from kaempferol and diethylenetriamine. Bioinorg. Chem. Appl. 2014:354138. 10.1155/2014/354138 25371657 PMC4209760

[B52] Weast R. C. Astle M. J. (1984). CRC Handbook of Chemistry and Physics, 64 Edn. Boca Raton, FL: CRC.

[B53] Wolfe A. Shimer G. H. Jr Meehan T. (1987). Polycyclic aromatic hydrocarbons physically intercalate into duplex regions of denatured DNA. Biochemistry 26, 6392–6396. 10.1021/bi00394a013 3427013

[B54] Woodruff D. N. Winpenny R. E. Layfield R. A. (2013). Lanthanide single-molecule magnets. Chem. Rev. 113, 5110–5148. 10.1021/cr400018q 23550940

[B55] Wu D. F. Liu Z. Ren P. Liu X. H. Wang N. Cui J. Z. (2019). A new family of dinuclear lanthanide complexes constructed from an 8-hydroxyquinoline Schiff base and β-diketone: magnetic properties and near-infrared luminescence. Dalton Trans. 48, 1392-1403, (b) Tang, Y., Feng, Y., Gan, X., Tan, M., Yu, K. (1996). Preparation and structure of [bis (8-quinolyloxyethyl)ether· H_3+_O]_3_[La(NO_3_)_6_]: A hexanitrato lanthanum complex of a hydronium ion podand complex cation. *Polyhedron* (1996). 15, 3219–3223. 10.1039/C8DT04384A

[B56] Yang L. Xu C. Ye W. Liu W. (2015). An electrochemical sensor for H_2_O_2_ based on a new Co-metal-organic framework modified electrode. Sens. Actuators B Chem. 215, 489–496. 10.1016/j.snb.2015.03.104

[B57] Yang Z. Y. Wang B. D. Li Y. H. (2006). Study on DNA-binding properties and cytotoxicity in L1210 of La (III) complex with PMBP-isonicotinoylhydrazone. J. Organomet. Chem. 691, 4159–4166. 10.1016/j.jorganchem.2006.06.002

[B58] Yang Z. Y. Yang R. D. Li F. S. Yu K. B. (2000). Crystal structure and antitumor activity of some rare earth metal complexes with Schiff base. Polyhedron 19, 2599–2604. 10.1016/S0277-5387(00)00562-3

[B59] Yuan S Qin J.-S. Lollar C. T. Zhou H.-C. (2018). Stable metal–organic frameworks with group 4 metals: current status and trends. ACS Cent. Sci. 4, 440–450. 10.1021/acscentsci.8b00073 29721526 PMC5920617

[B60] Zeng H. H. Qiu W. B. Zhang L. Liang R. P. Qiu J. D. (2016). Lanthanide coordination polymer nanoparticles as an excellent artificial peroxidase for hydrogen peroxide detection. Anal. Chem. 88, 6342–6348. 10.1021/acs.analchem.6b00630 27220993

[B61] Zeng H. H. Zhang L. Rong L. Q. Liang R. P. Qiu J. D. (2017). A luminescent lanthanide coordination polymer based on energy transfer from metal to metal for hydrogen peroxide detection. Biosens. Bioelectron. 89, 721–727. 10.1016/j.bios.2016.11.020 27865107

[B62] Zhang T. Manna K. Lin W. (2016). Metal–organic frameworks stabilize solution-inaccessible cobalt catalysts for highly efficient broad-scope organic transformations. J. Am. Chem. Soc. 138, 3241–3249. 10.1021/jacs.6b00849 26864496

[B63] Zhao W. Wang Z. Li Q. (2012). Fabrication of a nichrome electrode coated with silver microcrystals, and its application to sensing hydrogen peroxide. Anal. Methods. 4, 1105–1109. 10.1039/c2ay05904e

[B64] Zuber G. Quada J. C. Hecht S. M. (1998). Sequence selective cleavage of a DNA octanucleotide by chlorinated bithiazoles and bleomycins. J. Am. Chem. Soc. 120, 9368–9369. 10.1021/ja981937r

