# Supplementary material for: Single Molecule Magnets of Co2 and Co2La MOFs Synthesized by New Schiff Base Ligand N,N′-bis(o-Vanillinidene) Ethylenediamine (o-VEDH2)
Source: Front Chem. 2020 Nov 12;8:571223. doi: 10.3389/fchem.2020.571223 (PMC7689094; doi:10.3389/fchem.2020.571223)
Supplement: Supplementary file 1 [file Table_1.DOCX]

To

The Editor-in-Chief

Frontiers in Chemistry

Subject: Full APC Waiver

Manuscript ID: 571223

Manuscript Type: Article

Title: " **Single Molecule Magnets of Co_2_ and Co_2_La MOFs Synthesized by New Schiff base ligand N, N'-bis(*o*-vanillinidene)ethylenediamine (*o*-VEDH_2_)**"

Author(s): **Mithun Kumar Ghosh, Barun Jana, Tanmay Kumar Ghorai^*^**

Dear Sir,

Thanks for reviewing our research work for publication in your esteemed journal.

However, this is to bring your kind notice that the submitted research work was partially funded by MPCST, Madhya Pradesh, India. Our Institution (Indira Gandhi National Tribal University) was only given Fellowship to Mithun Kumar Ghosh (the first author of the manuscript) to perform this work. No analytical and publication budget head was granted by the funding agency as well as our institute.

Exemption from full APC will boost the morale and enthusiasm of poor research scholars (MKG, SP) and their future research endeavors.

The institute (IGNTU) is situated in a highly remote area, dominated by ethnic/tribal communities. The main focus of the University is to nurture indigenous population (Tribals) through teaching and social research.

The fellowship of the first author (MKG) was provided by Indira Gandhi National Tribal University, Amarkantak-484887, India. The funder never provided any support for open access publication and analytical expenses. Analysis and characterization (Single crystal XRD, Squid magnetic measurement & 1H NMR) of the complexes and ligand were done on request/free basis from IACS Kolkata and IISER Bhopal, Madhya Pradesh, India and Dr. Hari Singh Gour University (Central University), Madhya Pradesh, India by the personal pooled collection of authors.

Therefore, it is our humble request to kindly consider full APC waiver. We will be highly grateful to you for Full APC exemption.

Thanking you

Sincerely yours

Prof. Tanmay Kumar Ghorai

(Corresponding Author)
