# Supplementary material for: Single Molecule Magnets of Co2 and Co2La MOFs Synthesized by New Schiff Base Ligand N,N′-bis(o-Vanillinidene) Ethylenediamine (o-VEDH2)
Source: Front Chem. 2020 Nov 12;8:571223. doi: 10.3389/fchem.2020.571223 (PMC7689094; doi:10.3389/fchem.2020.571223)
Supplement: Supplementary file 2 [file Table_2.DOCX]

**Electronic Supplementary Information**

Single Molecule Magnets of Co_2_ and Co_2_La MOFs Synthesized by New Schiff base ligand N,N'-bis(*o*-vanillinidene) ethylenediamine (*o*-VEDH_2_)

**Mithun Kumar Ghosh^a^, Barun Jana^*c^ and Tanmay Kumar Ghorai^*ab^**

^a^Nanomaterials and Crystal Designing Laboratory, Department of Chemistry

Indira Gandhi National Tribal University, Amarkantak, M.P-484887, India

^b^Nanomaterials and Crystal Designing Laboratory, Department of Chemistry, Guru GhasidasVishwavidyalaya, Bilaspur (C. G.) – 495009, India

**^c^**Department of Chemistry, [Malaviya National Institute of Technology Jaipur](http://mnit.ac.in/),Rajasthan – 302017, India

**List of Contents:**

**Figure S1: ^1^HNMR of Schiff base ligand**

**Figure S2: MOF at Central projection, along 100 a axis of complex 1**

**ESI 3: Calculation of Exchange coupling constant (J) for complexes**

**Table S 1: Selected van dear Waal bond length (Å) for complex 1 and 2**

**Table: S2: Selected geometric information of the complex 1**

**Table S3: Selected geometric information of the complex 2**

**ESI 1. ^1^H NMR of the Schiff base ligand**

^1^H NMR (400 MHz, CDCl_3_) δ: 8.34 (s, 1H), 6.92 (d, J = 4 Hz, 1H), 6.87 (m, 1H), 6.82 (d, J = 8.3 Hz, 1H), 4.73 (s, J = 7.1 Hz, 1H), 3.97 (t, J = 12.1 Hz, 2H) and 3.50 (s, 3H)**^1^** shown in **Figure S1**

**
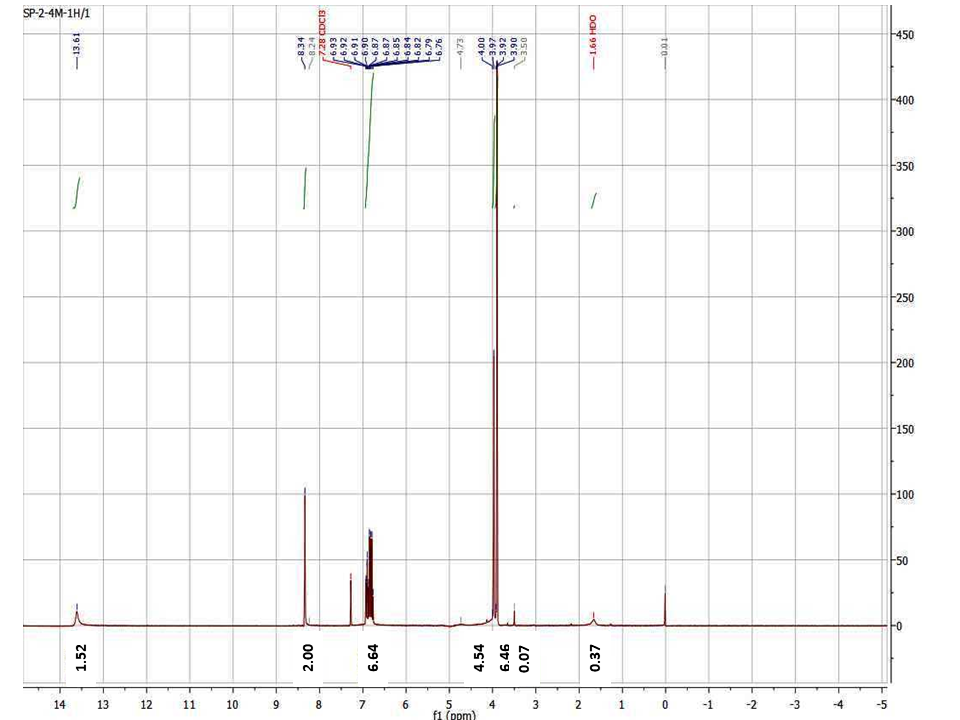

Figure S1: ^1^HNMR of Schiff base ligand**

**ESI 2: MOF size of complex 1**

MOF size: Side a; 2.4436 Å, b; 6.2205 Å and diagonal c; 5.8782 Å, d; 7.6012 Å


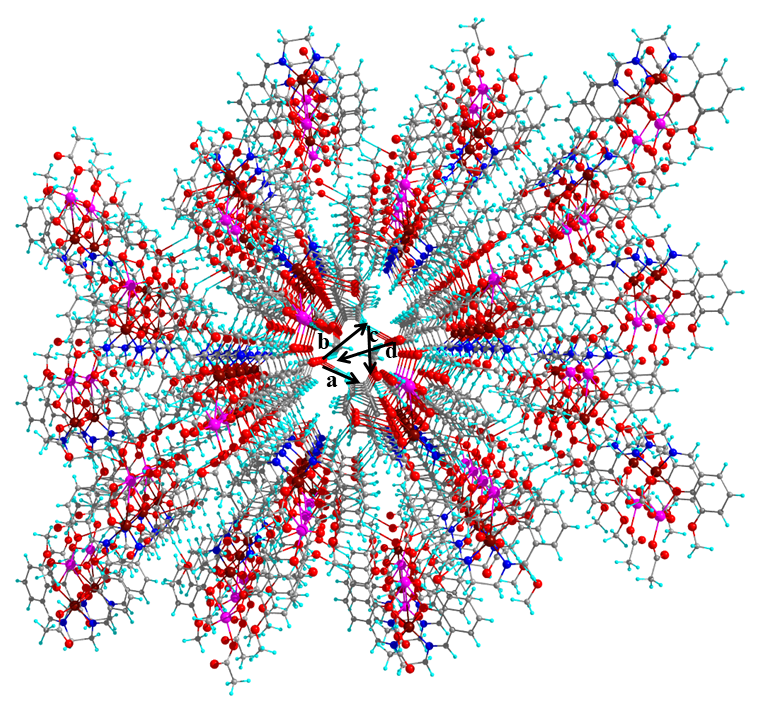


**Figure S2: MOF at Central projection, along 100 a axis of complex 1**

**ESI 3: Calculation of Exchange coupling constant (J) for complexes**

**Exchange coupling constant (J) for complexes** is calculated by using following equation

$$J=\frac{3\times Kb\times Tc}{2\times Z\times S(S+1)}$$

Where, Kb = Boltzmann constant, Tc = Currie temperature, Z = Number of atoms surrounding of unit cell and S for total ground state spin.

**Complex 1**

Kb = Boltzmann constant =8.617×10-5 eV K^-1^

Z =4; S= 4; Tc = 20K

S(S+1) = 4×5= 20

Putting all values and found the J value;

J = 3.231375 ×10^-5^ eV

J= 3.231375 ×10^-5^ × 8065.73 cm^-1^ 1eV = 8065.73 cm^-1^

= 26063.3982 ×10^-5^ cm^-1^

= 0.260 cm^-1^

**Complex 2**

Z = 4; S = 5; Tc = 5K

S(S+1) = 30

Putting all values and found the J value;

J = 0.53865 *10^-5^ eV

= 4343.89 *10^-5^ cm^-1^

= 0.043 cm^-1^

**Table S 1: Selected van dear Waal bond length (Å) for complex 1 and 2**

| **Complex 1** | | **Complex 2** | |
| --- | --- | --- | --- |
| **Bond Type** | **Bond length(Å)** | **Bond Type** | **Bond length(Å)** |
| O7-O11 | 2.523(114) | O1-O3 | 2.619(51) |
| O2-O6 | 2.605(97) | N9-O3 | 2.951(55) |
| C1-C16 | 2.395 (150) | N9-O4 | 2.982(53) |
| N2-N1 | 2.547(120) | H42B-O4 | 2.523(37) |
| N2-O4 | 2.617(106) | O1-O2 | 2.649(51) |
| O9-H00C | 2.412(80) | O2-O10 | 2.524(58) |
| N1-O1 | 2.764(105) | N7-O5 | 2.753(56) |
| O1-O3 | 2.811(101) | O5-O6 | 2.652(50) |

**Table S2: Selected geometric information of the complex 1**

| **Bond Type** | **Bond length(Å)** | **Bond Type** | **Bond length(Å)** |
| --- | --- | --- | --- |
| Co1—N2 | 1.856(8) | C15—H15B | 0.9800 |
| Co1—N1 | 1.876(8) | C15—H15C | 0.9800 |
| Co1—O8 | 1.892(7) | C8—C2 | 1.445(15) |
| Co1—O2 | 1.895(7) | C8—H8 | 0.9500 |
| Co1—O1 | 1.902(7) | C22—C23 | 1.512(16) |
| Co1—O4 | 1.920(7) | C16—H16 | 0.9500 |
| Co2—O10 | 2.004(8) | C17—H17A | 0.9800 |
| Co2—O7 | 2.065(8) | C17—H17B | 0.9800 |
| Co2—O3 | 2.062(8) | C17—H17C | 0.9800 |
| Co2—O1 | 2.069(7) | C19—H19A | 0.9800 |
| Co2—O2 | 2.137(7) | C19—H19B | 0.9800 |
| O2—C9 | 1.327(12) | C19—H19C | 0.9800 |
| O8—C18 | 1.303(13) | C20—C21 | 1.497(16) |
| O10—C22 | 1.277(14) | C3—C4 | 1.381(17) |
| O1—C1 | 1.338(12) | C3—C2 | 1.414(14) |
| O9—C18 | 1.238(14) | C3—H3 | 0.9500 |
| O12—C24 | 1.403(17) | C14—C13 | 1.382(15) |
| O12—H12 | 0.8400 | C11—C12 | 1.372(17) |
| N1—C8 | 1.280(14) | C11—H11 | 0.9500 |
| N1—C00K | 1.466(13) | C1—C6 | 1.409(14) |
| O6—C14 | 1.375(13) | C1—C2 | 1.411(15) |
| O6—C15 | 1.423(13) | C21—H21A | 0.9800 |
| O5—C6 | 1.379(13) | C21—H21B | 0.9800 |
| O5—C7 | 1.450(14) | C21—H21C | 0.9800 |
| O4—C20 | 1.285(13) | C24—H24A | 0.9801 |
| N2—C16 | 1.283(13) | C24—H24B | 0.9801 |
| N2—C00I | 1.476(13) | C24—H24C | 0.9801 |
| O3—C20 | 1.247(14) | C6—C5 | 1.379(16) |
| O11—C22 | 1.258(15) | C23—H23A | 0.9800 |
| O7—C17 | 1.428(14) | C23—H23B | 0.9800 |
| C10—C9 | 1.396(15) | C23—H23C | 0.9800 |
| C10—C11 | 1.404(15) | C12—C13 | 1.407(16) |
| C10—C16 | 1.446(15) | C12—H12A | 0.9500 |
| C00I—C00K | 1.517(16) | C13—H13 | 0.9500 |
| C00I—H00A | 0.9900 | C5—C4 | 1.411(18) |
| C00I—H00B | 0.9900 | C5—H5 | 0.9500 |
| C18—C19 | 1.504(17) | C4—H4 | 0.9500 |
| C00K—H00C | 0.9900 | C7—H7A | 0.9800 |
| C00K—H00D | 0.9900 | C7—H7B | 0.9800 |
| C9—C14 | 1.421(14) | C7—H7C | 0.9800 |
| C15—H15A | 0.9800 |  |  |
|  |  |  |  |
| **Bond Type** | **Angle (°)** | **Bond Type** | **Angle (°)** |
| N2—Co1—N1 | 86.0(4) | O11—C22—O10 | 124.8(10) |
| N2—Co1—O8 | 95.7(3) | O11—C22—C23 | 117.8(10) |
| N1—Co1—O8 | 92.5(3) | O10—C22—C23 | 117.4(11) |
| N2—Co1—O2 | 95.5(3) | N2—C16—C10 | 125.1(9) |
| N1—Co1—O2 | 178.4(3) | N2—C16—H16 | 117.400 |
| O8—Co1—O2 | 87.8(3) | C10—C16—H16 | 117.400 |
| N2—Co1—O1 | 178.0(3) | O7—C17—H17A | 109.500 |
| N1—Co1—O1 | 94.0(3) | O7—C17—H17B | 109.500 |
| O8—Co1—O1 | 86.4(3) | H17A—C17—H17B | 109.500 |
| O2—Co1—O1 | 84.4(3) | O7—C17—H17C | 109.500 |
| N2—Co1—O4 | 87.7(3) | H17A—C17—H17C | 109.500 |
| N1—Co1—O4 | 88.1(3) | H17B—C17—H17C | 109.500 |
| O8—Co1—O4 | 176.6(3) | C18—C19—H19A | 109.500 |
| O2—Co1—O4 | 91.4(3) | C18—C19—H19B | 109.500 |
| O1—Co1—O4 | 90.3(3) | H19A—C19—H19B | 109.500 |
| O10—Co2—O7 | 91.7(3) | C18—C19—H19C | 109.500 |
| O10—Co2—O3 | 88.4(3) | H19A—C19—H19C | 109.500 |
| O7—Co2—O3 | 173.3(3) | H19B—C19—H19C | 109.500 |
| O10—Co2—O1 | 156.9(3) | O3—C20—O4 | 126.8(10) |
| O7—Co2—O1 | 91.6(3) | O3—C20—C21 | 118.5(10) |
| O3—Co2—O1 | 85.8(3) | O4—C20—C21 | 114.7(10) |
| O10—Co2—O2 | 127.0(3) | C4—C3—C2 | 121.3(11) |
| O7—Co2—O2 | 100.8(3) | C4—C3—H3 | 119.400 |
| O3—Co2—O2 | 84.4(3) | C2—C3—H3 | 119.300 |
| O1—Co2—O2 | 74.6(3) | O6—C14—C13 | 125.1(9) |
| C9—O2—Co1 | 124.7(6) | O6—C14—C9 | 113.3(9) |
| C9—O2—Co2 | 137.3(6) | C13—C14—C9 | 121.6(10) |
| Co1—O2—Co2 | 97.8(3) | C12—C11—C10 | 121.7(10) |
| C18—O8—Co1 | 133.1(7) | C12—C11—H11 | 119.200 |
| C22—O10—Co2 | 128.8(7) | C10—C11—H11 | 119.200 |
| C1—O1—Co1 | 122.7(6) | O1—C1—C6 | 118.6(9) |
| C1—O1—Co2 | 120.6(6) | O1—C1—C2 | 121.9(9) |
| Co1—O1—Co2 | 100.0(3) | C6—C1—C2 | 119.5(9) |
| C24—O12—H12 | 109.500 | C20—C21—H21A | 109.500 |
| C8—N1—C00K | 120.2(9) | C20—C21—H21B | 109.500 |
| C8—N1—Co1 | 126.6(8) | H21A—C21—H21B | 109.500 |
| C00K—N1—Co1 | 113.2(7) | C20—C21—H21C | 109.500 |
| C14—O6—C15 | 116.3(8) | H21A—C21—H21C | 109.500 |
| C6—O5—C7 | 114.5(9) | H21B—C21—H21C | 109.500 |
| C20—O4—Co1 | 126.9(7) | O12—C24—H24A | 109.500 |
| C16—N2—C00I | 120.3(8) | O12—C24—H24B | 109.500 |
| C16—N2—Co1 | 126.0(7) | H24A—C24—H24B | 109.500 |
| C00I—N2—Co1 | 113.6(7) | O12—C24—H24C | 109.500 |
| C20—O3—Co2 | 128.6(7) | H24A—C24—H24C | 109.500 |
| C17—O7—Co2 | 126.8(7) | H24B—C24—H24C | 109.500 |
| C9—C10—C11 | 120.7(10) | C1—C2—C3 | 118.7(10) |
| C9—C10—C16 | 122.8(9) | C1—C2—C8 | 123.2(9) |
| C11—C10—C16 | 116.4(9) | C3—C2—C8 | 117.8(10) |
| N2—C00I—C00K | 106.6(8) | O5—C6—C5 | 124.9(10) |
| N2—C00I—H00A | 110.400 | O5—C6—C1 | 114.3(9) |
| C00K—C00I—H00A | 110.400 | C5—C6—C1 | 120.7(10) |
| N2—C00I—H00B | 110.400 | C22—C23—H23A | 109.500 |
| C00K—C00I—H00B | 110.400 | C22—C23—H23B | 109.500 |
| H00A—C00I—H00B | 108.600 | H23A—C23—H23B | 109.500 |
| O9—C18—O8 | 124.6(10) | C22—C23—H23C | 109.500 |
| O9—C18—C19 | 120.3(10) | H23A—C23—H23C | 109.500 |
| O8—C18—C19 | 115.(1) | H23B—C23—H23C | 109.500 |
| N1—C00K—C00I | 107.0(8) | C11—C12—C13 | 118.4(10) |
| N1—C00K—H00C | 110.300 | C11—C12—H12A | 120.800 |
| C00I—C00K—H00C | 110.300 | C13—C12—H12A | 120.800 |
| N1—C00K—H00D | 110.300 | C14—C13—C12 | 120.4(10) |
| C00I—C00K—H00D | 110.300 | C14—C13—H13 | 119.800 |
| H00C—C00K—H00D | 108.600 | C12—C13—H13 | 119.800 |
| O2—C9—C10 | 124.2(9) | C6—C5—C4 | 120.2(10) |
| O2—C9—C14 | 118.7(9) | C6—C5—H5 | 119.900 |
| C10—C9—C14 | 117.1(9) | C4—C5—H5 | 119.900 |
| O6—C15—H15A | 109.500 | C3—C4—C5 | 119.4(10) |
| O6—C15—H15B | 109.500 | C3—C4—H4 | 120.300 |
| H15A—C15—H15B | 109.500 | C5—C4—H4 | 120.300 |
| O6—C15—H15C | 109.500 | O5—C7—H7A | 109.500 |
| H15A—C15—H15C | 109.500 | O5—C7—H7B | 109.500 |
| H15B—C15—H15C | 109.500 | H7A—C7—H7B | 109.500 |
| N1—C8—C2 | 124.3(10) | O5—C7—H7C | 109.400 |
| N1—C8—H8 | 117.800 | H7A—C7—H7C | 109.500 |
| C2—C8—H8 | 117.800 | H7B—C7—H7C | 109.500 |

**Table S3: Selected geometric information of the complex 2**

| **Bond Type** | **Bond length(Å)** | **Bond Type** | **Bond length(Å)** |
| --- | --- | --- | --- |
| La1—O24 | 2.585(4) | C4—C5 | 1.422(7) |
| La1—O14 | 2.604(4) | C9—C8 | 1.418(8) |
| La1—O29 | 2.613(4) | C12—C13 | 1.353(8) |
| La1—O27 | 2.625(4) | C12—C11 | 1.415(7) |
| La1—O23 | 2.651(4) | C12—H12 | 0.9500 |
| La1—O26 | 2.651(4) | C10—C11 | 1.431(7) |
| La1—O21 | 2.652(4) | C10—H10 | 0.9500 |
| La1—O18 | 2.654(4) | C26—C27 | 1.425(7) |
| La1—O20 | 2.659(4) | C26—C25 | 1.434(7) |
| La1—O30 | 2.662(4) | C25—H25 | 0.9500 |
| La1—O17 | 2.663(4) | C21—C22 | 1.520(7) |
| La1—O15 | 2.749(4) | C21—H21A | 0.9900 |
| Co2—N7 | 1.876(4) | C21—H21B | 0.9900 |
| Co2—N6 | 1.884(4) | C34—C35 | 1.348(8) |
| Co2—O6 | 1.911(4) | C34—C33 | 1.415(7) |
| Co2—O5 | 1.914(4) | C34—H34 | 0.9500 |
| Co2—N8 | 1.950(4) | C8—C7 | 1.377(7) |
| Co2—O7 | 1.973(4) | C30—C29 | 1.386(7) |
| Co1—N1 | 1.878(5) | C22—H22A | 0.9900 |
| Co1—N2 | 1.889(4) | C22—H22B | 0.9900 |
| Co1—O2 | 1.901(4) | C13—C14 | 1.402(8) |
| Co1—O1 | 1.916(4) | C13—H13 | 0.9500 |
| Co1—N3 | 1.941(4) | C23—C24 | 1.494(8) |
| Co1—N4 | 1.944(5) | C23—H23A | 0.9900 |
| O1—C16 | 1.322(6) | C23—H23B | 0.9900 |
| O5—C31 | 1.321(6) | C24—H24A | 0.9900 |
| O15—N11 | 1.274(6) | C24—H24B | 0.9900 |
| O8—C30 | 1.374(6) | C43—C42 | 1.517(7) |
| O8—C39 | 1.444(6) | C43—H43A | 0.9900 |
| O4—C8 | 1.369(6) | C43—H43B | 0.9900 |
| O4—C17 | 1.426(6) | C7—C6 | 1.404(8) |
| O2—C9 | 1.326(6) | C7—H7 | 0.9500 |
| O6—C38 | 1.329(6) | C15—C14 | 1.385(7) |
| O9—C37 | 1.365(6) | C29—C28 | 1.400(8) |
| O9—C40 | 1.437(6) | C29—H29 | 0.9500 |
| O30—N16 | 1.271(6) | C14—H14 | 0.9500 |
| O14—N11 | 1.268(6) | C28—C27 | 1.361(8) |
| O7—C41 | 1.449(7) | C28—H28 | 0.9500 |
| O26—N15 | 1.256(6) | C36—C35 | 1.402(8) |
| O20—N13 | 1.249(6) | C36—H36 | 0.9500 |
| O32—N17 | 1.259(6) | C3—H3 | 0.9500 |
| O17—N12 | 1.270(6) | C42—H42A | 0.9900 |
| O21—N13 | 1.266(6) | C42—H42B | 0.9900 |
| O16—N11 | 1.219(6) | C35—H35 | 0.9500 |
| O3—C15 | 1.381(6) | C19—C20 | 1.466(8) |
| O3—C18 | 1.444(6) | C18—H18A | 0.9800 |
| N6—C32 | 1.287(7) | C18—H18B | 0.9800 |
| N6—C23 | 1.469(7) | C18—H18C | 0.9800 |
| O23—N14 | 1.276(7) | C5—C6 | 1.357(9) |
| O29—N16 | 1.272(6) | C5—H5 | 0.9500 |
| O18—N12 | 1.275(6) | C40—H40A | 0.9800 |
| O31—N16 | 1.225(6) | C40—H40B | 0.9800 |
| O27—N15 | 1.264(6) | C40—H40C | 0.9800 |
| O33—N17 | 1.248(6) | C27—H27 | 0.9500 |
| O34—N17 | 1.259(6) | C39—H39A | 0.9800 |
| O22—N13 | 1.240(6) | C39—H39B | 0.9800 |
| N8—C42 | 1.480(6) | C39—H39C | 0.9800 |
| N8—H8A | 0.9100 | C17—H17A | 0.9800 |
| N8—H8B | 0.9100 | C17—H17B | 0.9800 |
| N9—C43 | 1.498(7) | C17—H17C | 0.9800 |
| N3—C21 | 1.489(6) | C20—H20A | 0.9800 |
| N3—H3A | 0.9100 | C20—H20B | 0.9800 |
| N3—H3B | 0.9100 | C20—H20C | 0.9800 |
| N7—C25 | 1.291(7) | C41—H41A | 0.9800 |
| N7—C24 | 1.483(7) | C41—H41B | 0.9800 |
| N5—C22 | 1.472(6) | C41—H41C | 0.9800 |
| N2—C10 | 1.292(7) | C6—H6 | 0.9500 |
| N2—C1 | 1.464(7) | C45—N10 | 1.28(2) |
| N15—O28 | 1.222(7) | C45—C44 | 1.333(19) |
| O24—N14 | 1.250(7) | C44—H44A | 0.9800 |
| N1—C3 | 1.293(7) | C44—H44B | 0.9800 |
| N1—C2 | 1.457(9) | C44—H44C | 0.9800 |
| O25—N14 | 1.229(7) | C2—C1 | 1.321(10) |
| N4—C19 | 1.133(7) | C2—H2 | 0.9500 |
| O19—N12 | 1.218(6) | C1—H1 | 0.9500 |
| C31—C30 | 1.412(7) | O11—C47 | 1.401(8) |
| C31—C26 | 1.425(7) | O11—H11 | 0.8400 |
| C37—C36 | 1.378(7) | O12—C46 | 1.453(11) |
| C37—C38 | 1.424(7) | O12—H12A | 0.8400 |
| C16—C11 | 1.410(7) | C47—H47A | 0.9800 |
| C16—C15 | 1.414(7) | C47—H47B | 0.9800 |
| C32—C33 | 1.431(7) | C47—H47C | 0.9800 |
| C32—H32 | 0.9500 | C46—H46A | 0.9800 |
| C38—C33 | 1.409(7) | C46—H46B | 0.9800 |
| C4—C9 | 1.404(7) | C46—H46C | 0.9800 |
| C4—C3 | 1.421(8) |  |  |
| **Bond Type** | **Angle (°)** | **Bond Type** | **Angle (°)** |
| O24—La1—O14 | 175.53(14) | O25—N14—O24 | 121.1(6) |
| O24—La1—O29 | 65.99(13) | O25—N14—O23 | 121.9(6) |
| O14—La1—O29 | 110.64(13) | O24—N14—O23 | 117.0(5) |
| O24—La1—O27 | 68.90(14) | O25—N14—La1 | 170.4(5) |
| O14—La1—O27 | 107.52(12) | O24—N14—La1 | 57.5(3) |
| O29—La1—O27 | 71.89(14) | O23—N14—La1 | 60.6(3) |
| O24—La1—O23 | 48.55(14) | O6—C38—C33 | 124.7(5) |
| O14—La1—O23 | 133.13(14) | O6—C38—C37 | 117.9(5) |
| O29—La1—O23 | 110.73(14) | C33—C38—C37 | 117.4(5) |
| O27—La1—O23 | 66.46(13) | C9—C4—C3 | 122.2(5) |
| O24—La1—O26 | 110.94(14) | C9—C4—C5 | 120.4(5) |
| O14—La1—O26 | 64.72(12) | C3—C4—C5 | 117.4(5) |
| O29—La1—O26 | 70.08(13) | O2—C9—C4 | 124.7(5) |
| O27—La1—O26 | 47.96(12) | O2—C9—C8 | 117.9(5) |
| O23—La1—O26 | 111.21(13) | C4—C9—C8 | 117.4(5) |
| O24—La1—O21 | 71.91(13) | C13—C12—C11 | 121.1(5) |
| O14—La1—O21 | 110.10(12) | C13—C12—H12 | 119.400 |
| O29—La1—O21 | 70.17(14) | C11—C12—H12 | 119.400 |
| O27—La1—O21 | 133.63(14) | N2—C10—C11 | 125.4(5) |
| O23—La1—O21 | 103.85(13) | N2—C10—H10 | 117.300 |
| O26—La1—O21 | 133.96(13) | C11—C10—H10 | 117.300 |
| O24—La1—O18 | 117.68(14) | C27—C26—C31 | 120.4(5) |
| O14—La1—O18 | 65.79(14) | C27—C26—C25 | 117.4(5) |
| O29—La1—O18 | 175.97(13) | C31—C26—C25 | 122.1(5) |
| O27—La1—O18 | 110.66(13) | N7—C25—C26 | 124.7(5) |
| O23—La1—O18 | 73.29(14) | N7—C25—H25 | 117.600 |
| O26—La1—O18 | 109.08(13) | C26—C25—H25 | 117.600 |
| O21—La1—O18 | 108.91(13) | N3—C21—C22 | 111.3(4) |
| O24—La1—O20 | 73.77(14) | N3—C21—H21A | 109.400 |
| O14—La1—O20 | 110.59(13) | C22—C21—H21A | 109.400 |
| O29—La1—O20 | 113.65(13) | N3—C21—H21B | 109.400 |
| O27—La1—O20 | 135.43(13) | C22—C21—H21B | 109.400 |
| O23—La1—O20 | 70.82(13) | H21A—C21—H21B | 108.000 |
| O26—La1—O20 | 175.15(13) | C35—C34—C33 | 121.1(5) |
| O21—La1—O20 | 47.80(13) | C35—C34—H34 | 119.400 |
| O18—La1—O20 | 66.96(12) | C33—C34—H34 | 119.400 |
| O24—La1—O30 | 110.86(13) | O4—C8—C7 | 124.3(5) |
| O14—La1—O30 | 66.97(13) | O4—C8—C9 | 114.4(4) |
| O29—La1—O30 | 48.39(12) | C7—C8—C9 | 121.3(5) |
| O27—La1—O30 | 103.97(12) | O8—C30—C29 | 124.1(5) |
| O23—La1—O30 | 158.83(14) | O8—C30—C31 | 114.4(4) |
| O26—La1—O30 | 68.28(12) | C29—C30—C31 | 121.5(5) |
| O21—La1—O30 | 68.27(12) | C38—C33—C34 | 120.1(5) |
| O18—La1—O30 | 127.58(13) | C38—C33—C32 | 121.9(5) |
| O20—La1—O30 | 111.55(12) | C34—C33—C32 | 117.9(5) |
| O24—La1—O17 | 112.89(13) | N5—C22—C21 | 109.9(4) |
| O14—La1—O17 | 66.94(13) | N5—C22—H22A | 109.700 |
| O29—La1—O17 | 133.04(14) | C21—C22—H22A | 109.700 |
| O27—La1—O17 | 65.39(14) | N5—C22—H22B | 109.700 |
| O23—La1—O17 | 69.04(13) | C21—C22—H22B | 109.700 |
| O26—La1—O17 | 67.25(13) | H22A—C22—H22B | 108.200 |
| O21—La1—O17 | 156.74(14) | C16—C11—C12 | 120.7(5) |
| O18—La1—O17 | 48.09(13) | C16—C11—C10 | 122.3(5) |
| O20—La1—O17 | 110.21(13) | C12—C11—C10 | 117.0(5) |
| O30—La1—O17 | 125.75(12) | C12—C13—C14 | 119.3(5) |
| O24—La1—O15 | 135.64(13) | C12—C13—H13 | 120.300 |
| O14—La1—O15 | 47.55(11) | C14—C13—H13 | 120.300 |
| O29—La1—O15 | 108.51(12) | N6—C23—C24 | 106.8(5) |
| O27—La1—O15 | 154.43(12) | N6—C23—H23A | 110.400 |
| O23—La1—O15 | 132.08(13) | C24—C23—H23A | 110.400 |
| O26—La1—O15 | 107.21(11) | N6—C23—H23B | 110.400 |
| O21—La1—O15 | 65.48(12) | C24—C23—H23B | 110.400 |
| O18—La1—O15 | 67.80(13) | H23A—C23—H23B | 108.600 |
| O20—La1—O15 | 68.93(12) | N7—C24—C23 | 106.7(4) |
| O30—La1—O15 | 64.09(12) | N7—C24—H24A | 110.400 |
| O17—La1—O15 | 102.05(12) | C23—C24—H24A | 110.400 |
| N7—Co2—N6 | 84.88(19) | N7—C24—H24B | 110.400 |
| N7—Co2—O6 | 177.20(18) | C23—C24—H24B | 110.400 |
| N6—Co2—O6 | 94.15(17) | H24A—C24—H24B | 108.600 |
| N7—Co2—O5 | 93.15(17) | N9—C43—C42 | 107.9(4) |
| N6—Co2—O5 | 177.96(17) | N9—C43—H43A | 110.100 |
| O6—Co2—O5 | 87.79(15) | C42—C43—H43A | 110.100 |
| N7—Co2—N8 | 91.12(18) | N9—C43—H43B | 110.100 |
| N6—Co2—N8 | 88.91(18) | C42—C43—H43B | 110.100 |
| O6—Co2—N8 | 91.49(16) | H43A—C43—H43B | 108.400 |
| O5—Co2—N8 | 91.67(17) | C8—C7—C6 | 120.5(5) |
| N7—Co2—O7 | 90.31(18) | C8—C7—H7 | 119.700 |
| N6—Co2—O7 | 88.60(17) | C6—C7—H7 | 119.700 |
| O6—Co2—O7 | 87.04(16) | O3—C15—C14 | 123.9(5) |
| O5—Co2—O7 | 90.88(16) | O3—C15—C16 | 114.9(4) |
| N8—Co2—O7 | 177.01(17) | C14—C15—C16 | 121.2(5) |
| N1—Co1—N2 | 84.7(2) | C30—C29—C28 | 120.6(5) |
| N1—Co1—O2 | 93.56(18) | C30—C29—H29 | 119.700 |
| N2—Co1—O2 | 178.11(18) | C28—C29—H29 | 119.700 |
| N1—Co1—O1 | 177.3(2) | C15—C14—C13 | 120.7(5) |
| N2—Co1—O1 | 93.88(17) | C15—C14—H14 | 119.600 |
| O2—Co1—O1 | 87.89(15) | C13—C14—H14 | 119.600 |
| N1—Co1—N3 | 91.3(2) | C27—C28—C29 | 120.0(5) |
| N2—Co1—N3 | 89.18(19) | C27—C28—H28 | 120.000 |
| O2—Co1—N3 | 90.13(18) | C29—C28—H28 | 120.000 |
| O1—Co1—N3 | 90.95(17) | C37—C36—C35 | 120.4(5) |
| N1—Co1—N4 | 90.5(2) | C37—C36—H36 | 119.800 |
| N2—Co1—N4 | 91.0(2) | C35—C36—H36 | 119.800 |
| O2—Co1—N4 | 89.74(19) | N1—C3—C4 | 125.3(5) |
| O1—Co1—N4 | 87.21(19) | N1—C3—H3 | 117.300 |
| N3—Co1—N4 | 178.2(2) | C4—C3—H3 | 117.300 |
| C16—O1—Co1 | 125.8(3) | N8—C42—C43 | 111.1(4) |
| C31—O5—Co2 | 125.7(3) | N8—C42—H42A | 109.400 |
| N11—O15—La1 | 94.3(3) | C43—C42—H42A | 109.400 |
| C30—O8—C39 | 116.1(4) | N8—C42—H42B | 109.400 |
| C8—O4—C17 | 116.5(4) | C43—C42—H42B | 109.400 |
| C9—O2—Co1 | 126.4(3) | H42A—C42—H42B | 108.000 |
| C38—O6—Co2 | 126.1(3) | C34—C35—C36 | 120.0(5) |
| C37—O9—C40 | 116.6(4) | C34—C35—H35 | 120.000 |
| N16—O30—La1 | 95.8(3) | C36—C35—H35 | 120.000 |
| N11—O14—La1 | 101.6(3) | N4—C19—C20 | 178.3(7) |
| C41—O7—Co2 | 123.2(3) | O3—C18—H18A | 109.500 |
| N15—O26—La1 | 96.9(3) | O3—C18—H18B | 109.500 |
| N13—O20—La1 | 97.0(3) | H18A—C18—H18B | 109.500 |
| N12—O17—La1 | 97.2(3) | O3—C18—H18C | 109.500 |
| N13—O21—La1 | 96.9(3) | H18A—C18—H18C | 109.500 |
| C15—O3—C18 | 116.7(4) | H18B—C18—H18C | 109.500 |
| C32—N6—C23 | 118.9(4) | C6—C5—C4 | 120.7(5) |
| C32—N6—Co2 | 126.7(4) | C6—C5—H5 | 119.700 |
| C23—N6—Co2 | 114.2(3) | C4—C5—H5 | 119.700 |
| N14—O23—La1 | 94.6(3) | O9—C40—H40A | 109.500 |
| N16—O29—La1 | 98.2(3) | O9—C40—H40B | 109.500 |
| N12—O18—La1 | 97.5(3) | H40A—C40—H40B | 109.500 |
| N15—O27—La1 | 98.0(3) | O9—C40—H40C | 109.500 |
| C42—N8—Co2 | 117.3(3) | H40A—C40—H40C | 109.500 |
| C42—N8—H8A | 108.000 | H40B—C40—H40C | 109.500 |
| Co2—N8—H8A | 108.000 | C28—C27—C26 | 120.5(5) |
| C42—N8—H8B | 108.000 | C28—C27—H27 | 119.700 |
| Co2—N8—H8B | 108.000 | C26—C27—H27 | 119.700 |
| H8A—N8—H8B | 107.200 | O8—C39—H39A | 109.500 |
| C21—N3—Co1 | 116.0(3) | O8—C39—H39B | 109.500 |
| C21—N3—H3A | 108.300 | H39A—C39—H39B | 109.500 |
| Co1—N3—H3A | 108.300 | O8—C39—H39C | 109.500 |
| C21—N3—H3B | 108.300 | H39A—C39—H39C | 109.500 |
| Co1—N3—H3B | 108.300 | H39B—C39—H39C | 109.500 |
| H3A—N3—H3B | 107.400 | O4—C17—H17A | 109.500 |
| O16—N11—O14 | 121.3(5) | O4—C17—H17B | 109.500 |
| O16—N11—O15 | 122.2(5) | H17A—C17—H17B | 109.500 |
| O14—N11—O15 | 116.5(4) | O4—C17—H17C | 109.500 |
| C25—N7—C24 | 119.9(4) | H17A—C17—H17C | 109.500 |
| C25—N7—Co2 | 127.4(4) | H17B—C17—H17C | 109.500 |
| C24—N7—Co2 | 112.7(3) | C19—C20—H20A | 109.500 |
| C10—N2—C1 | 119.3(5) | C19—C20—H20B | 109.500 |
| C10—N2—Co1 | 127.1(4) | H20A—C20—H20B | 109.500 |
| C1—N2—Co1 | 113.4(4) | C19—C20—H20C | 109.500 |
| O31—N16—O30 | 121.9(5) | H20A—C20—H20C | 109.500 |
| O31—N16—O29 | 121.5(5) | H20B—C20—H20C | 109.500 |
| O30—N16—O29 | 116.5(5) | O7—C41—H41A | 109.500 |
| O31—N16—La1 | 169.6(4) | O7—C41—H41B | 109.500 |
| O30—N16—La1 | 59.8(3) | H41A—C41—H41B | 109.500 |
| O29—N16—La1 | 57.6(3) | O7—C41—H41C | 109.500 |
| O22—N13—O20 | 122.1(5) | H41A—C41—H41C | 109.500 |
| O22—N13—O21 | 120.2(5) | H41B—C41—H41C | 109.500 |
| O20—N13—O21 | 117.7(5) | C5—C6—C7 | 119.7(5) |
| O28—N15—O26 | 121.1(5) | C5—C6—H6 | 120.100 |
| O28—N15—O27 | 122.3(5) | C7—C6—H6 | 120.100 |
| O26—N15—O27 | 116.6(5) | N10—C45—C44 | 171.3(16) |
| O28—N15—La1 | 174.5(5) | C45—C44—H44A | 109.500 |
| O26—N15—La1 | 59.1(3) | C45—C44—H44B | 109.500 |
| O27—N15—La1 | 57.9(3) | H44A—C44—H44B | 109.500 |
| N14—O24—La1 | 98.4(3) | C45—C44—H44C | 109.500 |
| C3—N1—C2 | 120.4(5) | H44A—C44—H44C | 109.500 |
| C3—N1—Co1 | 127.6(4) | H44B—C44—H44C | 109.500 |
| C2—N1—Co1 | 112.0(4) | C1—C2—N1 | 116.0(7) |
| O33—N17—O32 | 120.6(5) | C1—C2—H2 | 122.000 |
| O33—N17—O34 | 121.1(5) | N1—C2—H2 | 122.000 |
| O32—N17—O34 | 118.4(5) | C2—C1—N2 | 112.4(6) |
| C19—N4—Co1 | 176.1(5) | C2—C1—H1 | 123.800 |
| O5—C31—C30 | 119.0(5) | N2—C1—H1 | 123.800 |
| O5—C31—C26 | 124.0(5) | C47—O11—H11 | 109.500 |
| C30—C31—C26 | 116.9(4) | C46—O12—H12A | 109.500 |
| O19—N12—O17 | 122.7(5) | O11—C47—H47A | 109.500 |
| O19—N12—O18 | 120.6(5) | O11—C47—H47B | 109.500 |
| O17—N12—O18 | 116.7(5) | H47A—C47—H47B | 109.500 |
| O9—C37—C36 | 123.9(5) | O11—C47—H47C | 109.500 |
| O9—C37—C38 | 115.2(4) | H47A—C47—H47C | 109.500 |
| C36—C37—C38 | 120.9(5) | H47B—C47—H47C | 109.500 |
| O1—C16—C11 | 125.1(5) | O12—C46—H46A | 109.500 |
| O1—C16—C15 | 118.0(5) | O12—C46—H46B | 109.500 |
| C11—C16—C15 | 116.9(5) | H46A—C46—H46B | 109.500 |
| N6—C32—C33 | 126.3(5) | O12—C46—H46C | 109.500 |
| N6—C32—H32 | 116.900 | H46A—C46—H46C | 109.500 |
| C33—C32—H32 | 116.900 | H46B—C46—H46C | 109.500 |

**Reference:**

1. Ghosh, M. K.; Pathak, S.; Ghorai, T. K. Synthesis of Two Mononuclear Schiff Base Metal (M= Fe, Cu) Complexes: MOF Structure, Dye Degradation, H_2_O_2_ Sensing, and DNA Binding Property. *ACS Omega*. **2019**, 4, 16068-16079.
