# Supplementary material for: Single Molecule Magnets of Co2 and Co2La MOFs Synthesized by New Schiff Base Ligand N,N′-bis(o-Vanillinidene) Ethylenediamine (o-VEDH2)
Source: Front Chem. 2020 Nov 12;8:571223. doi: 10.3389/fchem.2020.571223 (PMC7689094; doi:10.3389/fchem.2020.571223)

checkCIF/PLATON report

You have not supplied any structure factors. As a result the full set of tests cannot be run.

THIS REPORT IS FOR GUIDANCE ONLY. IF USED AS PART OF A REVIEW PROCEDURE FOR PUBLICATION, IT SHOULD NOT REPLACE THE EXPERTISE OF AN EXPERIENCED CRYSTALLOGRAPHIC REFEREE.

No syntax errors found. CIF dictionary Interpreting this report

Datablock: test1_a

```
Bond precision:  C-C = 0.0081 A          Wavelength=0.71073

Cell:            a=12.8836(11)     b=19.6476(17)      c=25.664(2)
                 alpha=90          beta=94.574(3)     gamma=90
Temperature:     100 K

                Calculated                 Reported
Volume          6475.7(9)                  6475.7(9)
Space group     P 21/n                     P 21/n
Hall group      -P 2yn                     -P 2yn
                C22 H25 Co N5 O4, C21 H27
Moiety formula  Co N4 O5, La N6 O18, N O3, ?
                C2 H3 N, 2
Sum formula     C47 H63 Co2 La N17 O33     C47 H58 Co2 La N17 O33
Mr              1650.91                    1645.87
Dx,g cm-3       1.693                      1.688
Z               4                          4
Mu (mm-1)       1.260                      1.260
F000            3356.0                     3336.0
F000'           3359.37
h,k,lmax        16,25,32                   16,25,32
Nref            14317                      14277
Tmin,Tmax       0.970,0.975                0.970,0.975
Tmin'           0.939

Correction method= # Reported T Limits: Tmin=0.970 Tmax=0.975
AbsCorr = NONE

Data completeness= 0.997          Theta(max)= 27.115

R(reflections)= 0.0565( 9755)     wR2(reflections)= 0.1762( 14277)

S = 0.888                  Npar= 912
```

The following ALERTS were generated. Each ALERT has the format
test-name_ALERT_alert-type_alert-level.
Click on the hyperlinks for more details of the test.

Alert level A

```
PLAT213_ALERT_2_A Atom C2             has ADP max/min Ratio .....        5.8 prolat
```

Author Response: In the ligand framework C2 has disorder and resides in two position. disorder treatment has not been performed.

Alert level B

```
PLAT220_ALERT_2_B NonSolvent   Resd 1  C   Ueq(max)/Ueq(min) Range        8.3 Ratio
PLAT241_ALERT_2_B High   'MainMol' Ueq as Compared to Neighbors of         C2 Check
PLAT306_ALERT_2_B Isolated Oxygen Atom (H-atoms Missing ?) .......        O10 Check
PLAT430_ALERT_2_B Short Inter D...A Contact  O1       ..N9       .       2.76 Ang.
                                                 x,y,z  =      1_555 Check
PLAT430_ALERT_2_B Short Inter D...A Contact  O2       ..N9       .       2.76 Ang.
                                                 x,y,z  =      1_555 Check
PLAT430_ALERT_2_B Short Inter D...A Contact  O5       ..N5       .       2.81 Ang.
                                                 x,y,z  =      1_555 Check
PLAT430_ALERT_2_B Short Inter D...A Contact  O6       ..N5       .       2.79 Ang.
                                                 x,y,z  =      1_555 Check
PLAT430_ALERT_2_B Short Inter D...A Contact  O7       ..O34      .       2.69 Ang.
                                                 x,y,z  =      1_555 Check
PLAT430_ALERT_2_B Short Inter D...A Contact  O10      ..O14      .       2.75 Ang.
                                                 x,y,z  =      1_555 Check
PLAT430_ALERT_2_B Short Inter D...A Contact  O32      ..N9       .       2.84 Ang.
                                     1/2-x,1/2+y,3/2-z  =      2_556 Check
```

Alert level C

```
RINTA01_ALERT_3_C  The value of Rint is greater than 0.12
            Rint given   0.145
PLAT041_ALERT_1_C Calc. and Reported SumFormula    Strings  Differ     Please Check
PLAT043_ALERT_1_C Calculated and Reported Mol. Weight Differ by ..       5.04 Check
PLAT068_ALERT_1_C Reported F000 Differs from Calcd (or Missing)...     Please Check
PLAT222_ALERT_3_C NonSolvent Resd 1  H   Uiso(max)/Uiso(min) Range        7.7 Ratio
PLAT242_ALERT_2_C Low    'MainMol' Ueq as Compared to Neighbors of         N1 Check
PLAT242_ALERT_2_C Low    'MainMol' Ueq as Compared to Neighbors of         C1 Check
PLAT244_ALERT_4_C Low    'Solvent' Ueq as Compared to Neighbors of        C45 Check
PLAT260_ALERT_2_C Large Average Ueq of Residue Including       N10      0.165 Check
PLAT342_ALERT_3_C Low Bond Precision on  C-C Bonds ...............    0.00812 Ang.
PLAT364_ALERT_2_C Short  C(sp3)-C(sp)  Bond  C44      - C45      .       1.33 Ang.
```

Alert level G

```
FORMU01_ALERT_2_G  There is a discrepancy between the atom counts in the
            _chemical_formula_sum and the formula from the _atom_site* data.
            Atom count from _chemical_formula_sum:C47 H58 Co2 La1 N17 O33
            Atom count from the _atom_site data:  C47 H63 Co2 La1 N17 O33
CELLZ01_ALERT_1_G Difference between formula and atom_site contents detected.
CELLZ01_ALERT_1_G ALERT: Large difference may be due to a
            symmetry error - see SYMMG tests
           From the CIF: _cell_formula_units_Z    4
           From the CIF: _chemical_formula_sum  C47 H58 Co2 La N17 O33
           TEST: Compare cell contents of formula and atom_site data
```

```
          atom    Z*formula  cif sites diff
          C        188.00    188.00    0.00
          H        232.00    252.00  -20.00
          Co         8.00      8.00    0.00
          La         4.00      4.00    0.00
          N         68.00     68.00    0.00
          O        132.00    132.00    0.00
PLAT007_ALERT_5_G Number of Unrefined Donor-H Atoms ..............          6 Report
PLAT020_ALERT_3_G The Value of Rint is Greater Than 0.12 .........      0.145 Report
PLAT066_ALERT_1_G Predicted and Reported Tmin&Tmax Range Identical          ? Check
PLAT083_ALERT_2_G SHELXL Second Parameter in WGHT  Unusually Large      33.52 Why ?
PLAT432_ALERT_2_G Short Inter X...Y Contact  O10      ..C10              2.98 Ang.
                                                  x,y,z  =      1_555 Check
PLAT432_ALERT_2_G Short Inter X...Y Contact  O16      ..C32              2.98 Ang.
                                      1/2-x,1/2+y,3/2-z  =      2_556 Check
PLAT432_ALERT_2_G Short Inter X...Y Contact  O16      ..C34              3.00 Ang.
                                      1/2-x,1/2+y,3/2-z  =      2_556 Check
PLAT432_ALERT_2_G Short Inter X...Y Contact  O25      ..C19              3.00 Ang.
                                            1-x,1-y,1-z  =      3_666 Check
PLAT432_ALERT_2_G Short Inter X...Y Contact  O28      ..C3               2.98 Ang.
                                                  x,y,z  =      1_555 Check
PLAT432_ALERT_2_G Short Inter X...Y Contact  O31      ..N17              2.82 Ang.
                                                  x,y,z  =      1_555 Check
PLAT432_ALERT_2_G Short Inter X...Y Contact  C10      ..C34              3.13 Ang.
                                                1+x,y,z  =      1_655 Check
PLAT432_ALERT_2_G Short Inter X...Y Contact  C12      ..C32              3.18 Ang.
                                                1+x,y,z  =      1_655 Check
PLAT794_ALERT_5_G Tentative Bond Valency for Co1       (III)     .       3.54 Info
PLAT794_ALERT_5_G Tentative Bond Valency for Co2       (III)     .       3.34 Info
PLAT883_ALERT_1_G No Info/Value for _atom_sites_solution_primary .     Please Do !
PLAT965_ALERT_2_G The SHELXL WEIGHT Optimisation has not Converged     Please Check
```

---

  1 **ALERT level A** = Most likely a serious problem - resolve or explain
 10 **ALERT level B** = A potentially serious problem, consider carefully
 11 **ALERT level C** = Check. Ensure it is not caused by an omission or oversight
 19 **ALERT level G** = General information/check it is not something unexpected

  7 ALERT type 1 CIF construction/syntax error, inconsistent or missing data
 26 ALERT type 2 Indicator that the structure model may be wrong or deficient
  4 ALERT type 3 Indicator that the structure quality may be low
  1 ALERT type 4 Improvement, methodology, query or suggestion
  3 ALERT type 5 Informative message, check

It is advisable to attempt to resolve as many as possible of the alerts in all categories. Often the minor alerts point to easily fixed oversights, errors and omissions in your CIF or refinement strategy, so attention to these fine details can be worthwhile. In order to resolve some of the more serious problems it may be necessary to carry out additional measurements or structure refinements. However, the purpose of your study may justify the reported deviations and the more serious of these should normally be commented upon in the discussion or experimental section of a paper or in the "special_details" fields of the CIF. checkCIF was carefully designed to identify outliers and unusual parameters, but every test has its limitations and alerts that are not important in a particular case may appear. Conversely, the absence of alerts does not guarantee there are no aspects of the results needing attention. It is up to the individual to critically assess their own results and, if necessary, seek expert advice.

Publication of your CIF in IUCr journals

A basic structural check has been run on your CIF. These basic checks will be run on all CIFs submitted for publication in IUCr journals (*Acta Crystallographica*, *Journal of Applied Crystallography*, *Journal of Synchrotron Radiation*); however, if you intend to submit to *Acta Crystallographica Section C* or *E* or *IUCrData*, you should make sure that full publication checks are run on the final version of your CIF prior to submission.

Publication of your CIF in other journals

Please refer to the *Notes for Authors* of the relevant journal for any special instructions relating to CIF submission.

PLATON version of 16/07/2020; check.def file version of 12/07/2020

Datablock test1_a - ellipsoid plot

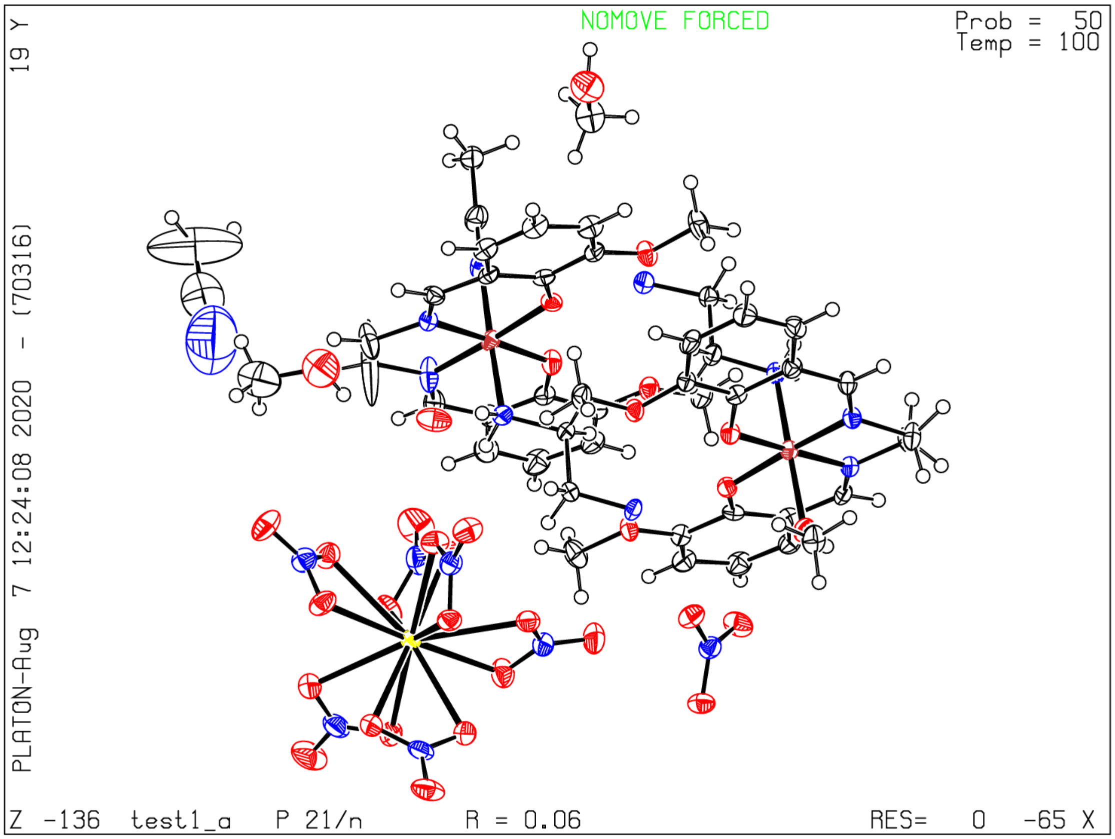

Supplement: Supplementary file 4 [file Data_Sheet_1.PDF]
