# Supplementary figures and images for: Single Molecule Magnets of Co2 and Co2La MOFs Synthesized by New Schiff Base Ligand N,N′-bis(o-Vanillinidene) Ethylenediamine (o-VEDH2)
Source: Front Chem. 2020 Nov 12;8:571223. doi: 10.3389/fchem.2020.571223 (PMC7689094; doi:10.3389/fchem.2020.571223)

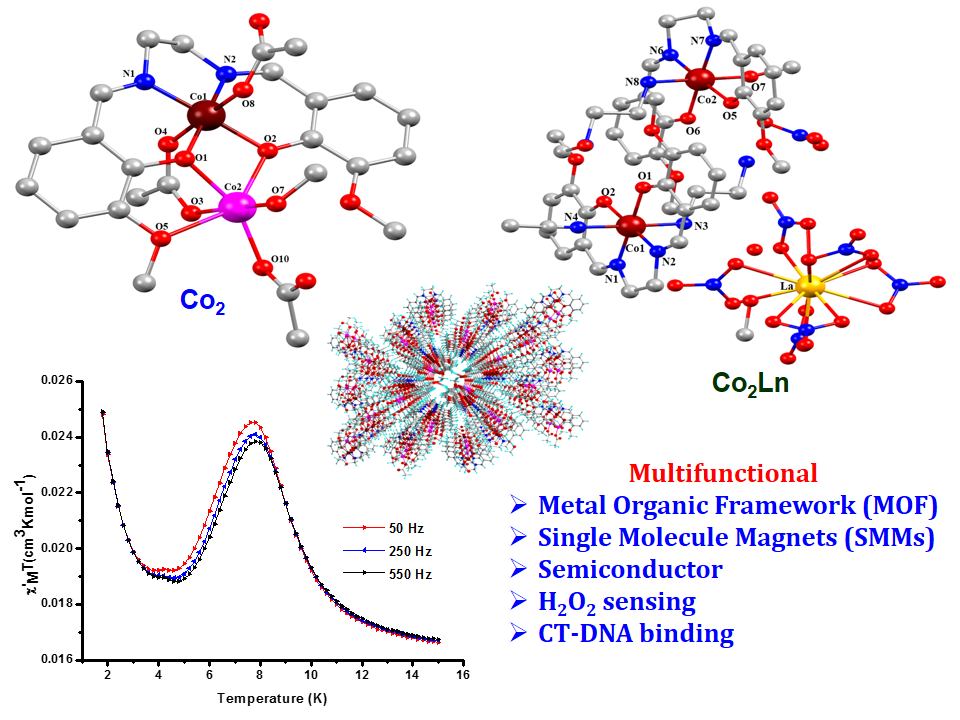

Supplement: Supplementary file 6 [file Image_1.TIF]
