# Supplementary material for: Single Molecule Magnets of Co2 and Co2La MOFs Synthesized by New Schiff Base Ligand N,N′-bis(o-Vanillinidene) Ethylenediamine (o-VEDH2)
Source: Front Chem. 2020 Nov 12;8:571223. doi: 10.3389/fchem.2020.571223 (PMC7689094; doi:10.3389/fchem.2020.571223)

checkCIF/PLATON report

You have not supplied any structure factors. As a result the full set of tests cannot be run.

THIS REPORT IS FOR GUIDANCE ONLY. IF USED AS PART OF A REVIEW PROCEDURE FOR PUBLICATION, IT SHOULD NOT REPLACE THE EXPERTISE OF AN EXPERIENCED CRYSTALLOGRAPHIC REFEREE.

No syntax errors found. CIF dictionary Interpreting this report

Datablock: mo_TANMOYAMARKANTAKCO26810_0m_a

```
Bond precision:  C-C = 0.0157 A          Wavelength=0.71073

Cell:            a=8.9157(14)     b=13.359(2)        c=24.066(4)
                 alpha=90         beta=90.602(6)     gamma=90
Temperature:     101 K

                Calculated                 Reported
Volume          2866.2(8)                  2866.2(8)
Space group     P 21/c                     P 21/c
Hall group      -P 2ybc                    -P 2ybc
Moiety formula  C25 H30 Co2 N2 O11, C H4 O ?
Sum formula     C26 H34 Co2 N2 O12         C26 H34 Co2 N2 O12
Mr              684.41                     684.41
Dx,g cm-3       1.586                      1.623
Z               4                          4
Mu (mm-1)       1.224                      1.192
F000            1416.0                     1764.0
F000'           1419.47
h,k,lmax        10,15,28                   10,15,28
Nref            5059                       4562
Tmin,Tmax       0.867,0.898
Tmin'           0.867

Correction method= Not given

Data completeness= 0.902          Theta(max)= 25.000

R(reflections)= 0.1239( 4271)     wR2(reflections)= 0.3538( 4562)

S = 1.260                  Npar= 388
```

The following ALERTS were generated. Each ALERT has the format
test-name_ALERT_alert-type_alert-level.
Click on the hyperlinks for more details of the test.

Alert level A

```
SHFSU01_ALERT_2_A  The absolute value of parameter shift to su ratio > 0.20
            Absolute value of the parameter shift to su ratio given   0.539
            Additional refinement cycles may be required.
```

Author Response: The structure has significant amount of disorder in the atomic positio Increasing the number of refinement cycle also does not improve the data

```
PLAT029_ALERT_3_A _diffrn_measured_fraction_theta_full value Low .      0.902 Why?
```

Author Response: The cystal was poorly diffracted at higher Bragg's angle. The situatio even after fresh recrystallization and data collection

```
PLAT080_ALERT_2_A Maximum Shift/Error ............................       0.54 Why ?
```

Author Response: Additional number of refinement cycle also does not improve the value.

Alert level B

```
PLAT341_ALERT_3_B Low Bond Precision on  C-C Bonds ...............    0.01572 Ang.
```

Author Response: This alert is generated because there is some amount of disorder in the structure

```
PLAT420_ALERT_2_B D-H Without Acceptor       O12      --H12      .     Please Check
```

Author Response: The value indicates that the interaction is very weak

---

Alert level C

```
ABSMU01_ALERT_1_C  The ratio of given/expected absorption coefficient lies
               outside the range 0.99 <> 1.01
            Calculated value of mu =     1.224
            Value of mu given      =     1.192
DENSD01_ALERT_1_C  The ratio of the submitted crystal density and that
            calculated from the formula is outside the range 0.99 <> 1.01
            Crystal density given    =      1.623
            Calculated crystal density =      1.586
PLAT046_ALERT_1_C Reported Z, MW and D(calc) are Inconsistent ....      1.586 Check
PLAT051_ALERT_1_C Mu(calc) and Mu(CIF) Ratio Differs from 1.0 by .       2.67 %
PLAT068_ALERT_1_C Reported F000 Differs from Calcd (or Missing)...     Please Check
PLAT082_ALERT_2_C High R1 Value ..................................       0.12 Report
PLAT084_ALERT_3_C High wR2 Value (i.e. > 0.25) ...................       0.35 Report
PLAT213_ALERT_2_C Atom N2             has ADP max/min Ratio .....        3.3 prolat
```

```
PLAT601_ALERT_2_C Unit Cell Contains Solvent Accessible VOIDS of .         35 Ang**3
```

Alert level G

```
PLAT007_ALERT_5_G Number of Unrefined Donor-H Atoms ..............          1 Report
PLAT072_ALERT_2_G SHELXL First  Parameter in WGHT  Unusually Large       0.18 Report
PLAT083_ALERT_2_G SHELXL Second Parameter in WGHT  Unusually Large      39.89 Why ?
PLAT720_ALERT_4_G Number of Unusual/Non-Standard Labels ..........          6 Note
PLAT794_ALERT_5_G Tentative Bond Valency for Co1       (III)     .       3.41 Info
PLAT794_ALERT_5_G Tentative Bond Valency for Co2       (II)      .       1.97 Info
PLAT802_ALERT_4_G CIF Input Record(s) with more than 80 Characters          2 Info
PLAT883_ALERT_1_G No Info/Value for _atom_sites_solution_primary .     Please Do !
PLAT941_ALERT_3_G Average HKL Measurement Multiplicity ...........        3.8 Low
PLAT965_ALERT_2_G The SHELXL WEIGHT Optimisation has not Converged     Please Check
```

```
  3 ALERT level A = Most likely a serious problem - resolve or explain
  2 ALERT level B = A potentially serious problem, consider carefully
  9 ALERT level C = Check. Ensure it is not caused by an omission or oversight
 10 ALERT level G = General information/check it is not something unexpected

  6 ALERT type 1 CIF construction/syntax error, inconsistent or missing data
  9 ALERT type 2 Indicator that the structure model may be wrong or deficient
  4 ALERT type 3 Indicator that the structure quality may be low
  2 ALERT type 4 Improvement, methodology, query or suggestion
  3 ALERT type 5 Informative message, check
```

It is advisable to attempt to resolve as many as possible of the alerts in all categories. Often the minor alerts point to easily fixed oversights, errors and omissions in your CIF or refinement strategy, so attention to these fine details can be worthwhile. In order to resolve some of the more serious problems it may be necessary to carry out additional measurements or structure refinements. However, the purpose of your study may justify the reported deviations and the more serious of these should normally be commented upon in the discussion or experimental section of a paper or in the "special_details" fields of the CIF. checkCIF was carefully designed to identify outliers and unusual parameters, but every test has its limitations and alerts that are not important in a particular case may appear. Conversely, the absence of alerts does not guarantee there are no aspects of the results needing attention. It is up to the individual to critically assess their own results and, if necessary, seek expert advice.

Publication of your CIF in IUCr journals

A basic structural check has been run on your CIF. These basic checks will be run on all CIFs submitted for publication in IUCr journals (*Acta Crystallographica*, *Journal of Applied Crystallography*, *Journal of Synchrotron Radiation*); however, if you intend to submit to *Acta Crystallographica Section C* or *E* or *IUCrData*, you should make sure that full publication checks are run on the final version of your CIF prior to submission.

Publication of your CIF in other journals

Please refer to the *Notes for Authors* of the relevant journal for any special instructions relating to CIF submission.

PLATON version of 16/07/2020; check.def file version of 12/07/2020

Datablock mo_TANMOYAMARKANTAKCO26810_0m_a - ellipsoid plot

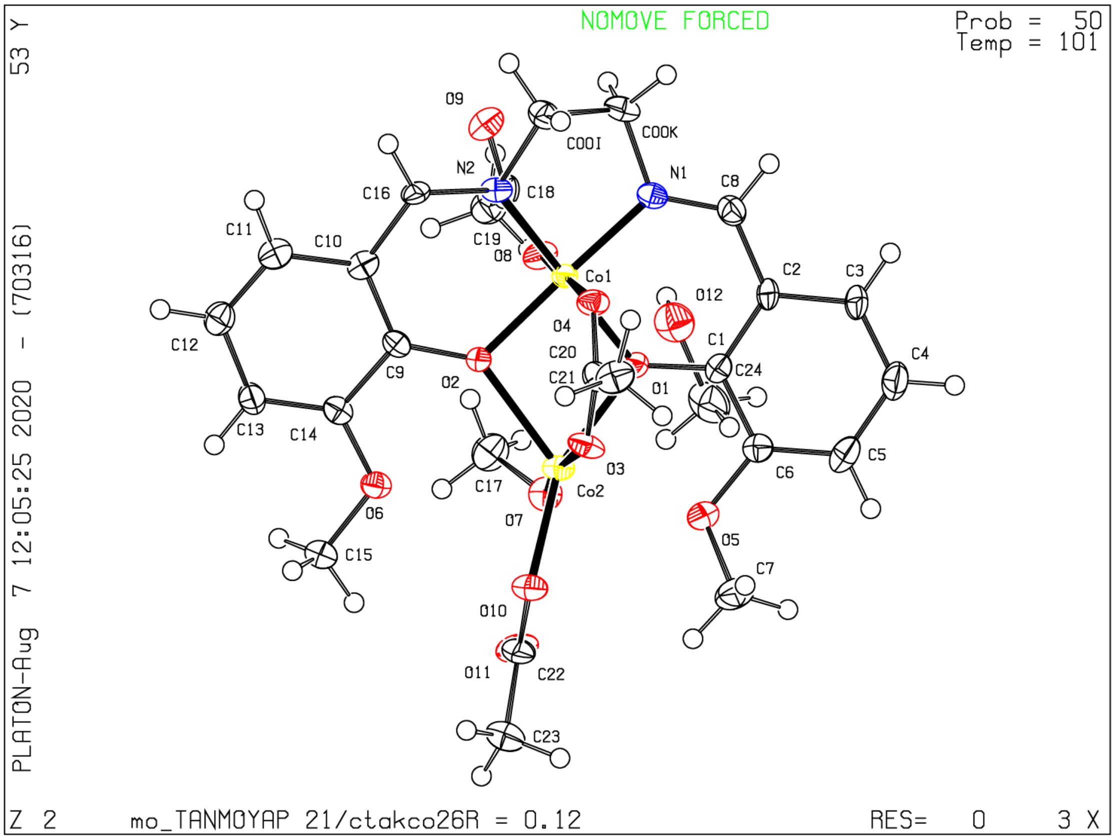

Supplement: Supplementary file 5 [file Data_Sheet_2.PDF]
