# Supplementary material for: Single Molecule Magnets of Co2 and Co2La MOFs Synthesized by New Schiff Base Ligand N,N′-bis(o-Vanillinidene) Ethylenediamine (o-VEDH2)
Source: Front Chem. 2020 Nov 12;8:571223. doi: 10.3389/fchem.2020.571223 (PMC7689094; doi:10.3389/fchem.2020.571223)
Supplement: Supplementary file 3 [file Table_3.DOCX]

**Response to the Reviewer’s comments**

**Reviewer # 2**: My initial doubts on this paper remain. I believe that in this form the manuscript cannot be published. These are my comments to the answers of the authors.

**Comments 1**: Magnetic study. I saw that, in the last version of the paper, the authors calculated the J values. I have three comments on this point:

**Comments 1 (i)**: The model used to calculate J must be included in the revision.

***Response****: As per the reviewer’s recommendation we have added the model equation which was used to calculate J values of complexes* ***1*** *and* ***2*** *in the supplementary information. Model equation and the calculations are as follows.*

**Exchange coupling constant (J) for complexes:**

$$J=\frac{3\times Kb\times Tc}{2\times Z\times S(S+1)}$$

**Complex 1**

Kb = Boltzmann constant =8.617×10^-5^ eV K^-1^

Z =4; S= 4; Tc = 20K

S(S+1) = 4×5= 20

Putting all values and found the J value;

J = 3.231375 ×10^-5^ eV

J= 3.231375 ×10^-5^ × 8065.73 cm^-1^	1eV = 8065.73 cm^-1^

= 26063.3982 ×10^-5^ cm^-1^

= 0.260 cm^-1^

**Complex 2**

Z = 4; S = 5; Tc = 5K

S(S+1) = 30

Putting all values and found the J value;

J = 0.53865 *10^-5^ eV

= 4343.89 *10-5 cm^-1^

= 0.043 cm^-1^

**Comments 1 (ii)**: The values of J are close to 0 and this is compatible with the structure of 2, where the Co ions are rather far, but not with 1, where they are close. The Co–Co distance should be added in the revision.

***Response****: First, we made some mistake in calculating the J values for complexes* ***1*** *and* ***2*** *as reported in our previous revised manuscript. After correcting our mistakes, we found J values of Complex* ***1*** *and* ***2*** *are 0.260 and 0.043 cm^-1^ respectively. The J value for complex* ***2*** *is in the line with our description. The J value for complex* ***1*** *is 0.260 which relatively low than the expected value but is in the acceptable limit. Hope the reviewer will agree with us.*

*We have already added the Co-Co distance in the revision manuscript shown in structural description page number 6 and line number 1.*

**Comments 1 (iii)**: J's ca. 0 are not compatible with the final spin state, which is the sum of the spins of Co1 and Co2 and which assumes that the interaction is strongly ferromagnetic.

***Response****: Yes, the newly calculated J value (0.260 cm^-1^) for complex* ***1*** *indicate a ferromagnetic interaction whereas J value (0.043 cm^-1^) for complex 2 indicate a antiferromagnetic interaction.*

**Comments 2**: Stability in aqueous solution. It is possible that the complexes are stable in solution, but this statement must be proved.

***Response****: Solubility of complexes* ***1*** *and* ***2*** *were checked in water and it was established that both of them are completely insoluble in water. This restrained us to perform the experiment in aqueous medium. Further, this led us to perform the UV-vis experiment in CH_3_CN, where the complexes are well soluble. Now, we hope the reviewer will understand the reason for choosing CH_3_CN instead of water as experiment medium.*

**Comments 3**: Reactions with H_2_O_2_. In the revised version of the manuscript, the authors write that OH· radicals are formed with 1 and not 2. This is possible but must be experimentally demonstrated. This is simply assumed. How did the authors prove this? This must also be provided in the experimental section.

***Response****: We understand the reviewer’s comments on our proposal of formation of .OH radical during the H_2_O_2_ reaction. Also, we understand that we do not have any direct evidence in support of our proposal. Just to mention that, here, we are not demonstrating the mechanism but we are only proposing a plausible mechanism which is very common in this kind of H_2_O_2_ reaction. Here, we are also surrendering that this time we will not be able to repeat the experiment in presence of some radical scavenger to establish our claim, because of COVID-19, our research lab and institutes are physically closed, only operating in online mode.*

*Just to inform, in our previous work, we demonstrated sensing property of a Fe complex against H_2_O_2_ which published ACS Omega 2019 (Ghosh et.al ACS Omega 4 (12), https://doi.org/10.1021/acsomega.9b02268).*

**Comments 4**: Experiments with CT-DNA.

I am not sure that the authors have demonstrated that a big complex like 2 is able to interact with DNA without any transformations. I don't see any structural models in the revision.

***Response****: Off course, this is very complicated to establish the actual interaction point between CT-DNA and complex* ***2*** *which would require large number of additional experiments along with in depth theoretical calculations. We think, current manuscript is out of scope for that work. Moreover, there are many research group working in this area. For example, Wright and co-workers documented the DNA binding property on various complexes those have bigger structure than complex* ***2*** *(Dalton Trans., 2015, 44, 3505).*
